# Supplementary material for: A nationwide school fruit and vegetable policy and childhood and adolescent overweight: A quasi-natural experimental study
Source: PLoS Med. 2022 Jan 18;19(1):e1003881. doi: 10.1371/journal.pmed.1003881 (PMC8765663; doi:10.1371/journal.pmed.1003881)
Supplement: S1 Checklist — (DOCX) [file pmed.1003881.s001.docx]

**S1 STROBE Statement - checklist of items included in “A nationwide school fruit and vegetable policy and childhood and adolescent overweight: A quasi-natural experimental study”.**

|  | Item No | Recommendation | Page  No |
| --- | --- | --- | --- |
| **Title and abstract** | 1 | (*a*) Indicate the study’s design with a commonly used term in the title or the abstract | Title |
|  |  | (*b*) Provide in the abstract an informative and balanced summary of what was done and what was found | Abstract |
| Introduction | | | |
| Background/rationale | 2 | Explain the scientific background and rationale for the investigation being reported | Introduction, paragraph 1-3. |
| Objectives | 3 | State specific objectives, including any prespecified hypotheses | Introduction, paragraph 3. |
| Methods | | | |
| Study design | 4 | Present key elements of study design early in the paper | Introduction, paragraph 3; and Methods, The free fruit and vegetable policy and analytical design, 1-2 paragraph. |
| Setting | 5 | Describe the setting, locations, and relevant dates, including periods of recruitment, exposure, follow-up, and data collection | Methods, The free fruit and vegetable policy and analytical design, paragraph 2; Methods, Data collection, paragraph 1-3; Method, Exposure classification, paragraph 1; and S1 Fig. |
| Participants | 6 | (*a*) *Cohort study*—Give the eligibility criteria, and the sources and methods of selection of participants. Describe methods of follow-up  *Case-control study*—Give the eligibility criteria, and the sources and methods of case ascertainment and control selection. Give the rationale for the choice of cases and controls  *Cross-sectional study*—Give the eligibility criteria, and the sources and methods of selection of participants | Methods, Participants, paragraph 1; and S3 Figure (participant flow charts). |
|  |  | (*b*) *Cohort study*—For matched studies, give matching criteria and number of exposed and unexposed  *Case-control study*—For matched studies, give matching criteria and the number of controls per case | NA |
| Variables | 7 | Clearly define all outcomes, exposures, predictors, potential confounders, and effect modifiers. Give diagnostic criteria, if applicable | Methods, Outcomes, paragraph 1; Methods, Exposure classification, paragraph 1; Methods, Free fruit and vegetable policy allocation and estimating a causal effect, paragraph 1; and S4 Text. |
| Data sources/ measurement | 8* | For each variable of interest, give sources of data and details of methods of assessment (measurement). Describe comparability of assessment methods if there is more than one group | Methods, Data collection, paragraph 1-3. |
| Bias | 9 | Describe any efforts to address potential sources of bias | Methods, Free fruit and vegetable policy allocation and estimating a causal effect, paragraph 1; S4 Text; S7 Text; S8 Text; and S5 Table. |
| Study size | 10 | Explain how the study size was arrived at | Results, paragraph 1; and S3 Fig. |
| Quantitative variables | 11 | Explain how quantitative variables were handled in the analyses. If applicable, describe which groupings were chosen and why | Methods, Outcomes, paragraph 1; Methods, Estimating the free fruit and vegetable policy effect, paragraph 1; S1 Text; and S3 Text. |
| Statistical methods | 12 | (*a*) Describe all statistical methods, including those used to control for confounding | Methods, Estimating the free fruit and vegetable policy effect, paragraph 1; Methods, Analyses, paragraph 1-4; and S3 Text. |
|  |  | (*b*) Describe any methods used to examine subgroups and interactions | Methods, Analyses, paragraph 2; |
|  |  | (*c*) Explain how missing data were addressed | S5 Table. |
|  |  | (*d*) *Cohort study*—If applicable, explain how loss to follow-up was addressed  *Case-control study*—If applicable, explain how matching of cases and controls was addressed  *Cross-sectional study*—If applicable, describe analytical methods taking account of sampling strategy | NA |
|  |  | (*e*) Describe any sensitivity analyses | Methods, The free fruit and vegetable policy and analytical design, paragraph 1-2; Methods, Analyses, paragraph 5; and S6 Text. |

| Results | | | |
| --- | --- | --- | --- |
| Participants | 13* | (a) Report numbers of individuals at each stage of study—eg numbers potentially eligible, examined for eligibility, confirmed eligible, included in the study, completing follow-up, and analysed | Results, paragraph 1, and S3 Figure. |
|  |  | (b) Give reasons for non-participation at each stage | S3 Fig |
|  |  | (c) Consider use of a flow diagram | S3 Fig |
| Descriptive data | 14* | (a) Give characteristics of study participants (eg demographic, clinical, social) and information on exposures and potential confounders | Results, paragraph 1; and S2 Table. |
|  |  | (b) Indicate number of participants with missing data for each variable of interest | S3 Fig. |
|  |  | (c) *Cohort study*—Summarise follow-up time (eg, average and total amount) | NA |
| Outcome data | 15* | *Cohort study*—Report numbers of outcome events or summary measures over time | S3 Fig |
|  |  | *Case-control study—*Report numbers in each exposure category, or summary measures of exposure | NA |
|  |  | *Cross-sectional study—*Report numbers of outcome events or summary measures | S3 Fig |
| Main results | 16 | (*a*) Give unadjusted estimates and, if applicable, confounder-adjusted estimates and their precision (eg, 95% confidence interval). Make clear which confounders were adjusted for and why they were included | Results, paragraph 4 and 10; Fig. 2; and Fig 4. |
|  |  | (*b*) Report category boundaries when continuous variables were categorized | NA |
|  |  | (*c*) If relevant, consider translating estimates of relative risk into absolute risk for a meaningful time period |  |
| Other analyses | 17 | Report other analyses done—eg analyses of subgroups and interactions, and sensitivity analyses | Results, paragraph 5-8 and paragraph 10; Fig 3; Fig 5; S6 Text; S5 Fig; S6 Fig; S8 Fig; S9 Fig; and S5 Table. |
| Discussion | | | |
| Key results | 18 | Summarise key results with reference to study objectives | Discussion, paragraph 1. |
| Limitations | 19 | Discuss limitations of the study, taking into account sources of potential bias or imprecision. Discuss both direction and magnitude of any potential bias | Discussion, paragraph 8-9; S7 Text; S8 Text; and S5 Table. |
| Interpretation | 20 | Give a cautious overall interpretation of results considering objectives, limitations, multiplicity of analyses, results from similar studies, and other relevant evidence | Discussion, paragraph 2-6; and S7 Text. |
| Generalisability | 21 | Discuss the generalisability (external validity) of the study results | Discussion, paragraph 8. |
| Other information | | | |
| Funding | 22 | Give the source of funding and the role of the funders for the present study and, if applicable, for the original study on which the present article is based | This work was supported by the Norwegian Research Council. |

*Give information separately for cases and controls in case-control studies and, if applicable, for exposed and unexposed groups in cohort and cross-sectional studies.

**Note:** An Explanation and Elaboration article discusses each checklist item and gives methodological background and published examples of transparent reporting. The STROBE checklist is best used in conjunction with this article (freely available on the Web sites of PLoS Medicine at http://www.plosmedicine.org/, Annals of Internal Medicine at http://www.annals.org/, and Epidemiology at http://www.epidem.com/). Information on the STROBE Initiative is available at www.strobe-statement.org.
