# Supplementary material for: A nationwide school fruit and vegetable policy and childhood and adolescent overweight: A quasi-natural experimental study
Source: PLoS Med. 2022 Jan 18;19(1):e1003881. doi: 10.1371/journal.pmed.1003881 (PMC8765663; doi:10.1371/journal.pmed.1003881)
Supplement: S1 Fig — The dashed square indicates the period with the FFV policy; the squares indicate measurements in the NCGS (2010, 2012, and 2015) and NYGS (2017); and the dots indicate approximate (routine) measurements included in analysis. FFV, free fruit and vegetable; NCGS, Norwegian Childhood Growth Study; NYGS, Norwegian Youth Growth Study. (DOCX) [file pmed.1003881.s002.docx]

**S1 Fig.**

**Supporting information - Study design**

**
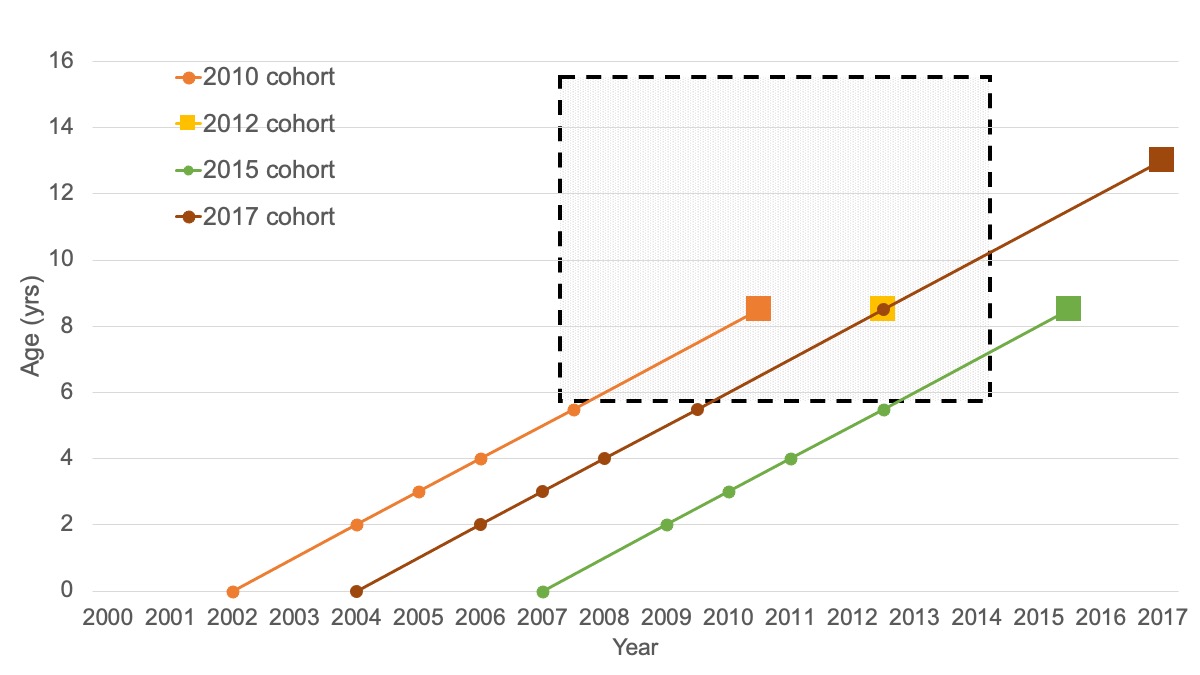
**

S1 Fig. Schematic of the quasi-natural experimental design. The dashed square indicates the period with the FFV policy; the squares indicate measurements in the NCGS (2010, 2012, and 2015) and NYGS (2017); and the dots indicate approximate (routine) measurements included in analysis.
FFV: Free fruit and vegetables; NCGS; Norwegian Childhood Growth Study; NYGS: Norwegian Youth Growth Study; yrs: years.
