# Supplementary material for: A nationwide school fruit and vegetable policy and childhood and adolescent overweight: A quasi-natural experimental study
Source: PLoS Med. 2022 Jan 18;19(1):e1003881. doi: 10.1371/journal.pmed.1003881 (PMC8765663; doi:10.1371/journal.pmed.1003881)
Supplement: S2 Fig — BMI, body mass index. (DOCX) [file pmed.1003881.s003.docx]

**S2 Fig.**

**Supporting information - Data Structure**





S2 Fig. Plot of individual values used in the analysis samples of BMI in each cohort (2010: orange; 2015: green; 2017: brown).

BMI: body mass index; yrs: years.
