# Supplementary material for: A nationwide school fruit and vegetable policy and childhood and adolescent overweight: A quasi-natural experimental study
Source: PLoS Med. 2022 Jan 18;19(1):e1003881. doi: 10.1371/journal.pmed.1003881 (PMC8765663; doi:10.1371/journal.pmed.1003881)
Supplement: S3 Fig — *Lost individuals are missing outcome. †Pre-intervention BMI adjusted model. Adj, adjusted; BMI, body mass index; Educ, parental education; FFV, free fruit and vegetable; NFFV, no free fruit and vegetable; pop-den, population density; WC, waist circumference. (DOCX) [file pmed.1003881.s004.docx]

**S3 Fig.**

**Supporting information – Flow charts**

S3 Fig. Participant flow charts by cohort.

^*^: lost individuals are missing outcome.

^†:^ Pre-intervention BMI adjusted model.

Adj: adjusted; BMI: body mass index; Educ: parental education; FFV: free fruit and vegetables; NFFV: no free fruit and vegetables (controls); pop-den: population density; WC: waist circumference; y: years.
