# Supplementary material for: A nationwide school fruit and vegetable policy and childhood and adolescent overweight: A quasi-natural experimental study
Source: PLoS Med. 2022 Jan 18;19(1):e1003881. doi: 10.1371/journal.pmed.1003881 (PMC8765663; doi:10.1371/journal.pmed.1003881)
Supplement: S4 Fig — The marginal proportions in each cohort and pooled cohorts, and in the crude and adjusted models, are presented. FFV, free fruit and vegetable; NFFV, no free fruit and vegetable. (DOCX) [file pmed.1003881.s005.docx]

# S4 Fig.

# Supporting information - Comparison of pre-intervention overweight/obesity trajectories
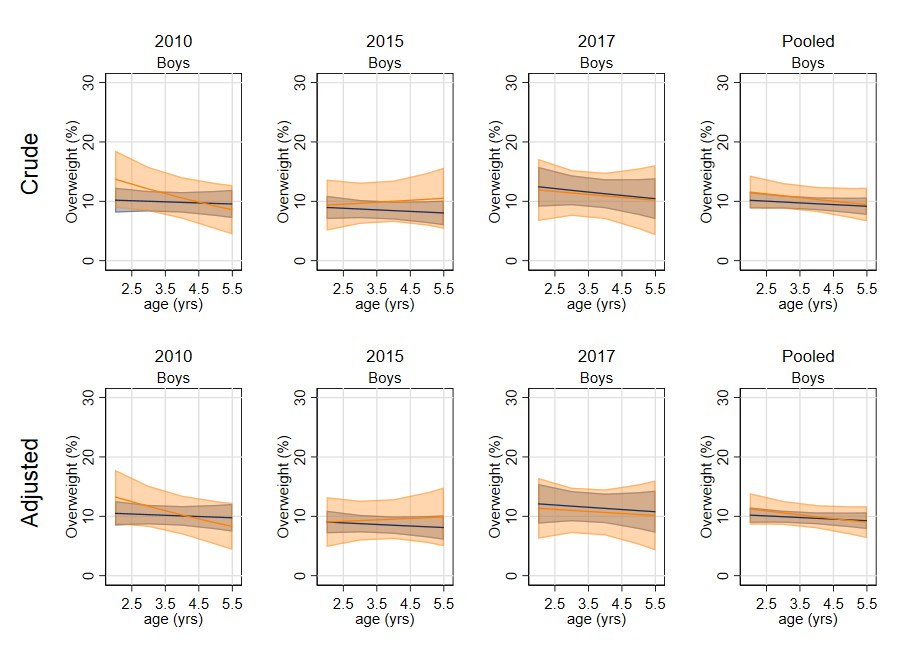

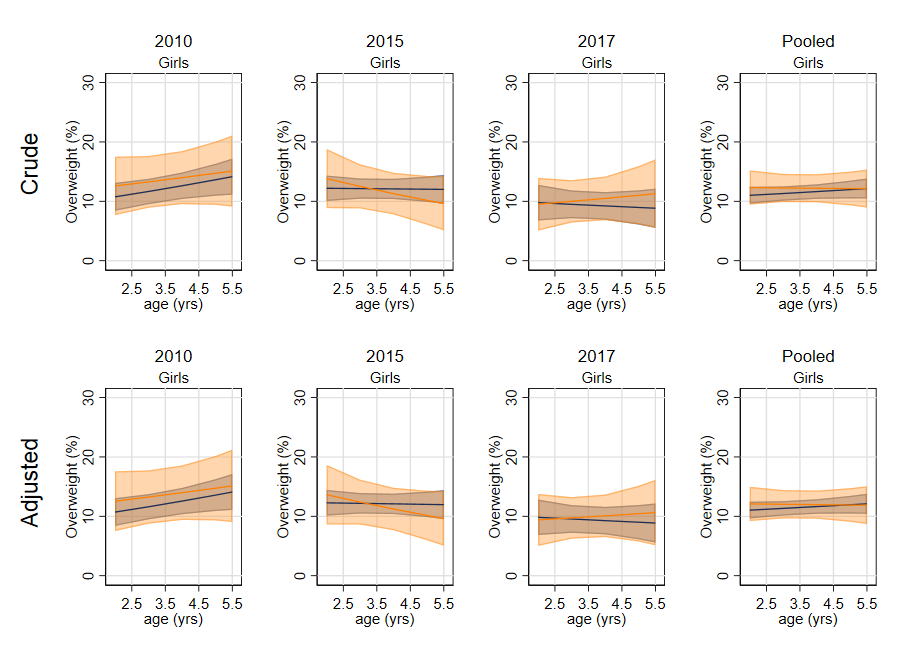


S4 Fig. Predicted pre-intervention (2 to 5.5 years) trajectories of overweight (including obesity) in boys and girls who would attend a FFV (orange) and a NFFV school (navy).

The marginal proportions in each cohort and pooled cohorts and in the crude and adjusted models are presented.

FFV: free fruit and vegetables; NFFV: no free fruit and vegetables (controls); yrs: years.
