# Supplementary material for: A nationwide school fruit and vegetable policy and childhood and adolescent overweight: A quasi-natural experimental study
Source: PLoS Med. 2022 Jan 18;19(1):e1003881. doi: 10.1371/journal.pmed.1003881 (PMC8765663; doi:10.1371/journal.pmed.1003881)
Supplement: S5 Fig — (a) BMISDS; (b) OW/OB; (c) WC; (d) WtHR. Results are presented by sex and cohort (including pooled) and for each model. Expressed as the difference in outcome or OR versus the counterfactual (as estimated using the NFFV schools) with 95% CI. Analysis of BMISDS and OW/OB: Pooled models include terms for cohort (intercept and slope). Adjusted models include region, population density, and highest parental education (all intercept and slope). +Pre-intervention adjusted models additionally include adjustment for BMISDS prior to the intervention. Note: Pre-intervention slopes were constrained to be the same in each group for all models except for BMISDS in 2015 cohort girls. Analysis of WC and WtHR: Outcomes are from grade 3 only. Pooled models include a term for cohort. Adjusted models include region, population density, and highest parental education. BMISDS, body mass index standard deviation score; FFV, free fruit and vegetable; NFFV, no free fruit and vegetable; OR, odds ratio; OW/OB, overweight and obesity; WC, waist circumference; WtHR, waist to height ratio. (DOCX) [file pmed.1003881.s006.docx]

# S5 Fig.

# Supporting information - Secondary/supplementary analyses

## Removal of no free fruit and vegetable schools (NFFV) that signed up to offer the parental paid fruit and vegetable subscription program at age 8.5 years

S5 Fig. Secondary analysis showing estimates of the FFV policy effect without NFFV schools that took part in the parental paid subscription program on (a) BMI_SDS_, (b) OW/OB, (c) WC, and (d) WtHR at 8.5 years.
Results are presented by sex and cohort (incl. pooled) and for each model. Expressed as the difference in outcome or odds ratio (OR) versus the counterfactual (as estimated using the NFFV schools) with 95% CI.

Analysis of BMI_SDS_ and OW/OB: Pooled models include terms for cohort (intercept and slope). Adjusted model includes region, population density, highest parental education (all intercept and slope). +Pre-intervention adjusted model additionally includes adjustment for BMI_SDS_ prior to the intervention. Note: Pre-intervention slopes were constrained to be the same in each group for all models except for BMI_SDS_ in cohort 2015 girls.
Analysis of WC and WtHR: Outcomes are from grade 3 only. Pooled models include a term for cohort. Adjusted model includes region, population density, highest parental education.

BMI_SDS_: body mass index standard deviation scores; CI: confidence interval; FFV: free fruit and vegetables; NFFV: no free fruit and vegetables; OR: odds ratio; OW/OB: overweight and obesity; WC: waist circumference; WtHR: waist to height ratio; y/yrs: year(s).
