# Supplementary material for: A nationwide school fruit and vegetable policy and childhood and adolescent overweight: A quasi-natural experimental study
Source: PLoS Med. 2022 Jan 18;19(1):e1003881. doi: 10.1371/journal.pmed.1003881 (PMC8765663; doi:10.1371/journal.pmed.1003881)
Supplement: S7 Fig — (a) BMISDS; (b) OW/OB; (c) WC; (d) WtHR. Results are presented by cohort (including pooled) and parental education for each model. Expressed as the difference in outcome or OR versus the counterfactual (as estimated using the NFFV schools without the subscription program) with 95% CI. The p-values are from a Wald test of the interaction between parental education and the subscription program. Analysis of BMISDS and OW/OB: Pooled models include terms for cohort (intercept and slope). Adjusted models include region and population density (all intercept and slope). +Pre-intervention adjusted models additionally include adjustment for BMISDS prior to the intervention. Analysis of WC and WtHR: Outcomes are from grade 3 only. Pooled models include a term for cohort. Adjusted models include region and population density. BMISDS, body mass index standard deviation score; NFFV, no free fruit and vegetable; OR, odds ratio; OW/OB, overweight and obesity; WC, waist circumference; WtHR, waist to height ratio. (DOCX) [file pmed.1003881.s008.docx]

# S7 Fig.

# Supporting information - Secondary/supplementary analyses

## Post-hoc analysis of the parental paid fruit and vegetable school subscription program among elementary schools only

S7 Fig. Estimates of the school fruit and vegetable subscription program (paid v no paid) on (a) BMI_SDS_, (b) OW/OB, (c) WC, and (d) WtHR at 8.5 years stratified by highest parental education level.

Results are presented cohort (incl. pooled) and parental education for each model. Expressed as the difference in outcome or odds ratio (OR) versus the counterfactual (as estimated using the NFFV schools without the subscription program) with 95% CI. The p-values are a Wald test of the interaction between parental education and the subscription program.

Analysis of BMI_SDS_ and OW/OB: Pooled models include terms for cohort (intercept and slope). Adjusted model includes region, population density (all intercept and slope). +Pre-intervention adjusted model additionally includes adjustment for BMI_SDS_ prior to the intervention.
Analysis of WC and WtHR: Outcomes are from grade 3 only. Pooled models include a term for cohort. Adjusted model includes region and population density.

BMI_SDS_: body mass index standard deviation scores; CI: confidence interval; NFFV: no free fruit or vegetable; OR: odds ratio; OW/OB: overweight and obesity; WC: waist circumference; WtHR: waist to height ratio; y/yrs: year(s).
