# Supplementary material for: A nationwide school fruit and vegetable policy and childhood and adolescent overweight: A quasi-natural experimental study
Source: PLoS Med. 2022 Jan 18;19(1):e1003881. doi: 10.1371/journal.pmed.1003881 (PMC8765663; doi:10.1371/journal.pmed.1003881)
Supplement: S8 Fig — (a) BMISDS; (b) OW/OB. Results are presented by sex for each model and expressed as the difference in outcome or OR versus the counterfactual at 13 years (as estimated using the NFFV schools) with 95% CI. Note that data are from the 2017 cohort only. Crude model has no adjustment. Adjusted models include region, population density, and highest parental education (intercept and slopes); +Pre-intervention adjusted models include additional adjustment for BMISDS prior to the intervention. BMISDS, body mass index standard deviation score; FFV, free fruit and vegetable; NFFV, no free fruit and vegetable; OR, odds ratio; OW/OB, overweight and obesity. (DOCX) [file pmed.1003881.s009.docx]

# S8 Fig.

# Supporting information - Secondary/supplementary analyses

Removal of NFFV schools that signed up to offer the parental paid fruit and vegetable subscription program at age 13 years

S8 Fig. Secondary analysis showing estimates of the FFV policy effect without NFFV schools that took part in the parental paid subscription program on (a) BMI_SDS_ and (b) OW/OB at age 13 years.

Results are presented by sex for each model and expressed as the difference in outcome or odds ratio (OR) versus the counterfactual at 13 years (as estimated using the NFFV schools) with 95% CI. Note data are from the 2017 cohort only.

Crude model: no adjustment. Adjusted model: includes region, population density, highest parental education (intercept and slopes); +Pre-intervention adjusted model: includes additional adjustment for BMI_SDS_ prior to the intervention.

BMI_SDS_: body mass index standard deviation scores; CI: confidence interval; FFV: free fruit and vegetables; NFFV: no free fruit or vegetable; OR: odds ratio; OW/OB: overweight and obesity; y: year(s).
