# Supplementary material for: A nationwide school fruit and vegetable policy and childhood and adolescent overweight: A quasi-natural experimental study
Source: PLoS Med. 2022 Jan 18;19(1):e1003881. doi: 10.1371/journal.pmed.1003881 (PMC8765663; doi:10.1371/journal.pmed.1003881)
Supplement: S9 Fig — (a) BMISDS; (b) OW/OB. Results are presented by sex and parental education for each model. Expressed as the difference in outcome or OR versus the counterfactual (as estimated using the NFFV schools) with 95% CI. The p-values are from a Wald test of the interaction between parental education and FFV. Note that data are from the 2017 cohort only. Adjusted models include terms for region and population density (intercept and slopes). +Pre-intervention adjusted models include additional adjustment for BMISDS prior to the intervention. BMISDS, body mass index standard deviation score; FFV, free fruit and vegetable; NFFV, no free fruit and vegetable; OR, odds ratio; OW/OB, overweight and obesity. (DOCX) [file pmed.1003881.s010.docx]

# S9 Fig.

# Supporting information - Secondary/supplementary analyses

Removal of NFFV schools that signed up to offer the parental paid fruit and vegetable subscription program at age 13 years

S9 Fig. Secondary analysis showing estimates of the FFV policy effect without NFFV schools that took part in the parental paid subscription program on (a) BMI_SDS_ and (b) OW/OB at age 13 years stratified by highest parental education level.

Results are presented by sex and parental education for each model. Expressed as the difference in outcome or odds ratio (OR) versus the counterfactual (as estimated using the NFFV schools) with 95% CI. The p-values are a Wald test of the interaction between parental education and FFV. Note data are from the 2017 cohort only.
Adjusted model includes terms for region and population density (intercept and slopes). +Pre-intervention adjusted model includes additional adjustment for BMI_SDS_ prior to the intervention.

BMI_SDS_: body mass index standard deviation scores; CI: confidence interval; FFV: free fruit and vegetables; NFFV: no free fruit and vegetables; OR: odds ratio; OW/OB: overweight and obesity; y: year(s).
