# Supplementary material for: A nationwide school fruit and vegetable policy and childhood and adolescent overweight: A quasi-natural experimental study
Source: PLoS Med. 2022 Jan 18;19(1):e1003881. doi: 10.1371/journal.pmed.1003881 (PMC8765663; doi:10.1371/journal.pmed.1003881)
Supplement: S1 Table — †Region and county at recruitment. FFV, free fruit and vegetable; NFFV, no free fruit and vegetable; Obs, observations. (DOCX) [file pmed.1003881.s011.docx]

**S1 Table**

**Supporting information - Data Structure**

S1 Table. Frequencies of schools, children, and observations by county illustrating the hierarchical data structure of the three longitudinal cohorts (pooled) based on the analysis sample.

|  |  | FFV | | | NFFV | | | All | | | |
| --- | --- | --- | --- | --- | --- | --- | --- | --- | --- | --- | --- |
| Region^†^ | County^†^ | N Schools | N Child | N Obs | N Schools | N Child | N Obs | N Schools | N Child | N Obs |  |
| Southern / Eastern | Akershus | 1 | 5 | 11 | 18 | 1270 | 3433 | 19 | 1275 | 3444 |  |
|  | Oslo | 5 | 218 | 559 | 8 | 352 | 915 | 13 | 570 | 1474 |  |
|  | Hedmark | 1 | 1 | 2 | 0 | 0 | 0 | 1 | 1 | 2 |  |
|  | Oppland | 0 | 0 | 0 | 1 | 1 | 2 | 1 | 1 | 2 |  |
|  | Vestfold | 9 | 131 | 305 | 20 | 712 | 1799 | 29 | 843 | 2104 |  |
|  | Telemark | 0 | 0 | 0 | 1 | 11 | 28 | 1 | 11 | 28 |  |
|  | Vest-Agder | 8 | 123 | 343 | 23 | 1007 | 2835 | 31 | 1130 | 3178 |  |
| Western | Rogaland | 7 | 257 | 797 | 23 | 691 | 2098 | 30 | 948 | 2895 |  |
|  | Hordaland | 10 | 154 | 400 | 23 | 754 | 2174 | 33 | 908 | 2574 |  |
| Central | Møre og Romsdal | 8 | 102 | 264 | 21 | 330 | 921 | 29 | 432 | 1185 |  |
|  | Sør-Trøndelag | 13 | 213 | 614 | 14 | 401 | 1127 | 27 | 614 | 1741 |  |
|  | Nord-Trøndelag | 0 | 4 | 11 | 1 | 5 | 14 | 1 | 9 | 25 |  |
| Northern | Nordland | 13 | 248 | 662 | 17 | 476 | 1372 | 30 | 724 | 2034 |  |
|  | Troms | 13 | 186 | 439 | 10 | 158 | 383 | 23 | 344 | 822 |  |
|  | Total | 88 | 1642 | 4407 | 180 | 6168 | 17101 | 268 | 7810 | 21508 |  |

^†^Region and county at recruitment.

FFV: free fruit and vegetables; NFFV: no free fruit and vegetables (controls); Obs: observations.
