# Supplementary material for: A nationwide school fruit and vegetable policy and childhood and adolescent overweight: A quasi-natural experimental study
Source: PLoS Med. 2022 Jan 18;19(1):e1003881. doi: 10.1371/journal.pmed.1003881 (PMC8765663; doi:10.1371/journal.pmed.1003881)
Supplement: S2 Table — *NFFV: Individuals who did not attend a school with FFV provision. FFV ≥ 1 year: Individuals who attended a school with FFV provision at least 1 year. ‡In third grade. †Of individuals attending NFFV schools, proportion who attended a school offering the paid fruit and vegetable subscription program. §Parental education prior to possible exposure (when the child was 4 years old). ªThese cohorts had longitudinal data and were pooled in the analysis of BMISDS and OW/OB. BMISDS, body mass index standard deviation score; FFV, free fruit and vegetable; NA, not applicable; NFFV, no free fruit and vegetable; OW/OB, overweight and obesity; Paid-sub, individuals attending schools offering the parental paid subscription program; SD, standard deviation. (DOCX) [file pmed.1003881.s012.docx]

**S2 Table**

**Supporting information – Description of individuals**

S2 Table. Description of individuals included in the analysis of outcomes at age 8.5 (third grade) by attendance at a FFV school in each cohort and pooled across cohorts.

|  | **2010 cohort** | | | **2012 cohort** | | | **2015 cohort** | | | **2017 cohort** | | | **Pooledª 2010, 2015 and 2017*** | | |
| --- | --- | --- | --- | --- | --- | --- | --- | --- | --- | --- | --- | --- | --- | --- | --- |
|  | Total | NFFV^*^ | FFV ≥ 1y^*^ | Total | NFFV^*^ | FFV ≥ 1y^*^ | Total | NFFV^*^ | FFV ≥ 1y^*^ | Total | NFFV^*^ | FFV ≥ 1y^*^ | Total | NFFV^*^ | FFV ≥ 1y^*^ |
| N (%) | 3125 | 2504 (80.1) | 621 (19.9) | 3405 | 2705 (79.4) | 700 (20.6) | 3207 | 2632 (82.1) | 575 (17.9) | 1478 | 1032 (69.8) | 446 (30.2) | 7810 | 6168 (79.0) | 1642 (21.0) |
| Paid-sub^†^, n (%) | 2505 | 830 (33.2) | NA | 2705 | 768 (28.4) | NA | 2632 | 696 (26.4) | NA | 1032 | 496 (48.1) | NA | 6168 | 2022 (32.8) | NA |
| Age (y), mean (SD)^‡^ | 8.3 (0.3) | 8.3 (0.3) | 8.3 (0.3) | 8.3 (0.3) | 8.3 (0.3) | 8.3 (0.3) | 8.3 (0.3) | 8.3 (0.3) | 8.3 (0.3) | 8.5 (0.4) | 8.5 (0.4) | 8.5 (0.4) | 8.3 (0.3) | 8.3 (0.3) | 8.4 (0.3) |
| Sex, n (%) |  |  |  |  |  |  |  |  |  |  |  |  |  |  |  |
| Boys | 1605 (51.4) | 1281 (51.2) | 324 (52.2) | 1755 (51.5) | 1380 (51.0) | 375 (53.6) | 1636 (51.0) | 1342 (51.0) | 296 (51.1) | 732 (49.5) | 518 (50.2) | 214 (48.0) | 3973 (50.9) | 3141 (50.9) | 832 (50.7) |
| Girls | 1520 (48.6) | 1223 (48.8) | 297 (47.8) | 1650 (48.5) | 1325 (49.0) | 325 (46.4) | 1571 (49.0) | 1290 (49.0) | 281 (48.9) | 746 (50.5) | 514 (49.8) | 232 (52.0) | 3837 (49.1) | 3027 (49.1) | 810 (49.3) |
| Population density, n (%)^‡^ | |  |  |  |  |  |  |  |  |  |  |  |  |  |  |
| Urban | 2445 (78.2) | 2033 (81.2) | 412 (66.3) | 2658 (78.1) | 2231 (82.5) | 427 (61.0) | 2486 (77.5) | 2134 (81.1) | 352 (61.2) | 897 (60.7) | 669 (64.8) | 228 (51.1) | 5828 (74.6) | 4836 (78.4) | 992 (60.4) |
| Semi-urban | 408 (13.1) | 301 (12.0) | 107 (17.2) | 456 (13.4) | 312 (11.5) | 144 (20.6) | 480 (15.0) | 350 (13.3) | 130 (22.6) | 360 (24.4) | 242 (23.5) | 118 (26.5) | 1248 (16.0) | 893 (14.5) | 355 (21.6) |
| Rural | 272 (8.7) | 170 (6.8) | 102 (16.4) | 291 (8.6) | 162 (6.0) | 129 (18.4) | 241 (7.5) | 148 (5.6) | 93 (16.2) | 221 (15.0) | 121 (11.7) | 100 (22.4) | 734 (9.4) | 439 (7.1) | 295 (18.0) |
| Region, n (%)^‡^ |  |  |  |  |  |  |  |  |  |  |  |  |  |  |  |
| Southern/Eastern | 1717 (54.9) | 1483 (59.2) | 234 (37.7) | 1868 (54.9) | 1627 (60.2) | 241 (34.4) | 1741 (54.3) | 1588 (60.3) | 153 (26.6) | 387 (26.1) | 284 (27.5) | 103 (23.1) | 3845 (49.2) | 3355 (54.4) | 490 (29.8) |
| Western | 706 (22.6) | 563 (22.5) | 143 (23.0) | 747 (21.9) | 570 (21.1) | 177 (25.3) | 748 (23.3) | 571 (21.7) | 177 (30.8) | 403 (27.3) | 311 (30.1) | 92 (20.6) | 1857 (23.8) | 1445 (23.4) | 412 (25.1) |
| Central | 365 (11.7) | 267 (10.7) | 98 (15.8) | 418 (12.3) | 298 (11.0) | 120 (17.1) | 346 (10.8) | 248 (9.4) | 98 (17.0) | 334 (22.6) | 220 (21.3) | 114 (25.6) | 1045 (13.4) | 735 (11.9) | 310 (18.9) |
| Northern | 337 (10.8) | 191 (7.6) | 146 (23.5) | 372 (10.9) | 210 (7.8) | 162 (23.1) | 372 (11.6) | 225 (8.6) | 147 (25.6) | 354 (24.0) | 217 (21.0) | 137 (30.7) | 1063 (13.6) | 633 (10.3) | 430 (26.2) |
| Parental education, n (%)^§^ | |  |  |  |  |  |  |  |  |  |  |  |  |  |  |
| <Higher | 1444 (46.2) | 1117 (44.6) | 327 (52.7) | 1431 (42.0) | 1119 (41.4) | 312 (44.6) | 1229 (38.3) | 973 (37.0) | 256 (44.5) | 586 (39.7) | 396 (38.4) | 190 (42.6) | 3259 (41.7) | 2486 (40.3) | 773 (47.1) |
| Higher+ | 1681 (53.8) | 1387 (55.4) | 294 (47.3) | 1974 (58.0) | 1586 (58.6) | 388 (55.4) | 1978 (61.7) | 1659 (63.0) | 319 (55.5) | 892 (60.4) | 636 (61.6) | 256 (57.4) | 4551 (58.3) | 3682 (59.7) | 869 (52.9) |

^*^NFFV: Individuals who did not attend a school with free fruit and vegetable provision; FFV ≥ 1y: Individuals attended a school with free fruit and vegetable provision at least one year.

^‡^In 3^rd^ grade, school

^†^Of in individuals attending NFFV schools, proportion who attend a school offering the paid fruit and vegetable subscription program.

^§^Parental education prior to possible exposure (when the child was four years old).

ªThese cohorts had longitudinal data and were pooled in analysis of BMI_SDS_ and OW/OB.

BMI_SDS_: body mass index standard deviation scores; FFV: Free fruit and vegetables; NA: not applicable; NFFV: No free fruit and vegetables; OW/OB: overweight and obesity; Paid-sub: Individuals attending schools offering the parental paid subscription program; SD: Standard deviation.; y: year.
