# Supplementary material for: A nationwide school fruit and vegetable policy and childhood and adolescent overweight: A quasi-natural experimental study
Source: PLoS Med. 2022 Jan 18;19(1):e1003881. doi: 10.1371/journal.pmed.1003881 (PMC8765663; doi:10.1371/journal.pmed.1003881)
Supplement: S3 Table — Differences in slope from 2 to 5.5 years and in BMISDS at 5.5 years in each cohort and pooled cohorts, and in the crude and adjusted models, are presented. †Crude pooled models include adjustment for cohort (intercept and slope). All models include a random intercept for school and random coefficients for child. ‡Adjusted models include region, population density, and highest parental education (intercept and slope); pooled adjusted models also include terms for cohort (intercept and slope). All models include random intercepts for school and random coefficients for child. aDifference in slope (BMISDS per year): FFV minus NFFV. bDifference in BMISDS at 5.5 years: FFV minus NFFV. BMISDS, body mass index standard deviation score; FFV, free fruit and vegetable; NFFV, no free fruit and vegetable. (DOCX) [file pmed.1003881.s013.docx]

**S3 Table**

**Supporting information - Comparison of pre-intervention BMI trajectories**

S3 Table. Estimated differences in pre-intervention (2 to 5.5 years) trajectories of BMI_SDS_ in boys and girls who would attend a FFV and a NFFV school.

|  |  | Crude^†^ | |  |  | Adjusted^‡^ |  |  |  |
| --- | --- | --- | --- | --- | --- | --- | --- | --- | --- |
|  | Cohort | Difference in slope ^(a)^  (95% CI) | p | Difference at 5.5y ^(b)^  (95% CI) | p | Difference in slope ^(a)^  (95% CI) | p | Difference at 5.5y ^(b)^  (95% CI) | p |
| Boys | 2010 | 0.007  (-0.044, 0.059) | 0.78 | 0.08  (-0.11, 0.26) | 0.43 | -0.001  (-0.05, 0.05) | 0.97 | 0.03  (-0.16, 0.22) | 0.76 |
|  | 2015 | 0.007  (-0.05, 0.06) | 0.8 | 0.15  (-0.04, 0.33) | 0.115 | -0.003  (-0.06, 0.05) | 0.92 | 0.13  (-0.05, 0.31) | 0.17 |
|  | 2017 | -0.014  (-0.076, 0.049) | 0.67 | 0.014  (-0.18, 0.21) | 0.89 | -0.02 (-0.08, 0.05) | 0.57 | -0.004  (-0.20, -0.19) | 0.97 |
|  | Pooled | 0.002  (-0.03, 0.03) | 0.89 | 0.08  (-0.03, 0.19) | 0.14 | -0.008  (-0.04, 0.024) | 0.63 | 0.05  (-0.06, 0.16) | 0.37 |
| Girls | 2010 | -0.000  (-0.05, 0.05) | 0.99 | 0.04  (-0.161, 0.241) | 0.69 | 0.00  (-0.05, 0.05) | 0.99 | 0.04  (-0.17, 0.24) | 0.73 |
|  | 2015 | -0.074  (-0.124, -0.023) | 0.004 | -0.195  (-0.37, -0.02) | 0.03 | -0.07  (-0.12, -0.02) | 0.006 | -0.21  (-0.39, -0.035) | 0.019 |
|  | 2017 | 0.026  (-0.034, 0.087) | 0.39 | 0.05  (-0.14, 0.24) | 0.63 | 0.015  (-0.05, 0.08) | 0.64 | -0.014  (-0.20, 0.18) | 0.88 |
|  | Pooled | -0.021  (-0.052, 0.010) | 0.188 | -0.05  (-0.161, 0.068) | 0.43 | -0.03  (-0.06, 0.007) | 0.12 | -0.08  (-0.20, 0.034) | 0.17 |

Differences in slope from 2 to 5.5 years and in BMI_SDS_ at 5.5 years in each cohort and pooled cohorts and in the crude and adjusted models are presented.

^†^Crude pooled model includes adjustment for cohort (intercept and slope). All models include a random intercept for school and random coefficients for child.

^‡^Adjusted model includes region, population density, highest parental education (intercept and slope); pooled adjusted model also includes terms for cohort (intercept and slope). All models include random intercepts for school and random coefficients for child.

^(a)^Difference in slope (BMI_SDS_ per year): FFV minus NFFV

^(b)^Difference in BMI_SDS_ at 5.5y: FFV minus NFFV

BMI_SDS_: body mass index standard deviation scores; CI: confidence interval; FFV: free fruit and vegetables; NFFV: no free fruit and vegetables; y: years.
