# Supplementary material for: A nationwide school fruit and vegetable policy and childhood and adolescent overweight: A quasi-natural experimental study
Source: PLoS Med. 2022 Jan 18;19(1):e1003881. doi: 10.1371/journal.pmed.1003881 (PMC8765663; doi:10.1371/journal.pmed.1003881)
Supplement: S4 Table — The ORs compare the slopes of the log odds of OW/OB from age 2 to 5.5 years and the odds of OW/OB at age 5.5 years (pre-intervention age). †Crude pooled models include adjustment for cohort (intercept and slope). All models include a random intercept for school and child. ‡Adjusted models include region, population density, and highest parental education (intercept and slope); pooled adjusted models also include terms for cohort (intercept and slope). All models include random intercepts for school and child. aOR comparing slopes of log odds (log odds per year) of overweight: FFV/NFFV. bOR comparing log odds of overweight at 5.5 years (pre-intervention): FFV/NFFV. FFV, free fruit and vegetable; NFFV, no free fruit and vegetable; OR, odds ratio. (DOCX) [file pmed.1003881.s014.docx]

**S4 Table**

**Supporting information - Comparison of pre-intervention overweight/obesity trajectories**

S4 Table. Odds ratios comparing pre-intervention (age 2 to 5.5 years) trajectories of overweight including obesity in boys and girls who would attend a FFV and a NFFV school.

|  |  | Crude† |  |  |  | Adjusted‡ |  |  |  |
| --- | --- | --- | --- | --- | --- | --- | --- | --- | --- |
|  | Cohort | OR per year ^(a)^  (95% CI) | p | OR at 5.5y ^(b)^  (95% CI) | p | OR per year ^(a)^  (95% CI) | p | OR at 5.5y ^(b)^  (95% CI) | p |
| Boys | 2010 | 0.79  (0.55, 1.13) | 0.20 | 0.81  (0.28, 2.34) | 0.7 | 0.79  (0.55, 1.14) | 0.21 | 0.73  (0.26, 2.05) | 0.54 |
|  | 2015 | 1.13  (0.75, 1.71) | 0.56 | 1.67  (0.56, 4.97) | 0.35 | 1.11  (0.74, 1.69) | 0.61 | 1.46  (0.49, 4.36) | 0.49 |
|  | 2017 | 1.01  (0.63, 1.63) | 0.96 | 0.95  (0.27, 3.32) | 0.94 | 1.00  (0.62, 1.63) | 0.99 | 0.89  (0.25, 3.16) | 0.86 |
|  | Pooled | 0.94  (0.75, 1.20) | 0.64 | 1.06  (0.55, 2.03) | 0.9 | 0.93  (0.73, 1.18) | 0.55 | 0.95  (0.50, 1.80) | 0.87 |
| Girls | 2010 | 0.94  (0.63, 1.39) | 0.76 | 1.18  (0.36, 3.84) | 0.78 | 0.94  (0.65, 1.40) | 0.77 | 1.20  (0.36, 3.98) | 0.77 |
|  | 2015 | 0.81  (0.54, 3.30) | 0.29 | 0.62  (0.21, 1.83) | 0.39 | 0.82  (0.55, 1.22) | 0.33 | 0.63  (0.21, 1.88) | 0.41 |
|  | 2017 | 1.17  (0.71, 1.91) | 0.53 | 1.64  (0.45, 5.93) | 0.45 | 1.14  (0.69, 1.89) | 0.60 | 1.45  (0.38, 5.48) | 0.58 |
|  | Pooled | 0.93  (0.73, 1.18) | 0.55 | 1.00  (0.50, 1.97) | 0.99 | 0.93  (0.73, 1.18) | 0.56 | 0.96  (0.48, 1.91) | 0.9 |

The ORs compare the slopes of the log odds of OW/OB from age 2 to 5.5 years and the odds of OW/OB at age 5.5 years (pre-intervention age).

† Crude pooled model includes adjustment for cohort (intercept and slope). All models include a random intercept for school and child.

‡ Adjusted model includes region, population density, highest parental education (intercept and slope); pooled adjusted model also includes terms for cohort (intercept and slope). All models include random intercepts for school and child.

^(a)^ OR comparing slopes of log odds (log odds per year) of overweight: FFV/NFFV

^(b)^ OR comparing log odds of overweight at 5.5y (pre-intervention): FFV/NFFV

CI: confidence interval; FFV: free fruit and vegetables; NFFV: no free fruit and vegetables; OR: odds ratio; y: years.
