# Supplementary material for: A nationwide school fruit and vegetable policy and childhood and adolescent overweight: A quasi-natural experimental study
Source: PLoS Med. 2022 Jan 18;19(1):e1003881. doi: 10.1371/journal.pmed.1003881 (PMC8765663; doi:10.1371/journal.pmed.1003881)
Supplement: S5 Table — (DOCX) [file pmed.1003881.s015.docx]

**S5 Table**

**Supporting information - Summary of analyses designed to test robustness of results and assess consistency of evidence**

S5 Table. Summary of some of the analyses that were performed to check the robustness and consistency of results.

| **Component** | **Target check** | **Potential direction and/or cause of bias** | **Analysis/ steps to combat/check** | **Results** |
| --- | --- | --- | --- | --- |
| Analytical strategy | Parameterization of longitudinal models | Unknown.  Sensitivity of findings to bias caused by suboptimal model of functional form. | Re-ran analyses with an earlier (5.3y) and later (5.7y) knot point. | Findings unaltered – results available on request |
| Analysis strategy | Scale for modelling the BMI outcome | Unknown  Suboptimal model fit | Used sex and age internally standardized BMI. | Described in main text and supporting information  Improved the residual diagnostics compared the raw scale or externally standardized scale |
| Analysis strategy | Missing outcome and exposure data | Unknown  (1) Outcomes in children missing exposure or covariables are MNAR  (2) Participation in study linked to outcome – MNAR  (3) Studies are retrospective cohorts, so bias could also be caused by missing pre-intervention data being MNAR. | NB; main longitudinal models give unbiased estimates from the missingness if the missing data are MAR  (1) Compare estimates in the unadjusted models when using complete cases and all available data  (2) & (3) Differences in exclusions due to missing data between group may provide an indication. | Described in main text  (1) Findings unaltered – results available on request  (2) & (3) Proportions missing similar between groups – although this does not exclude MNAR. |
| Confounding | Residual confounding | Away from null  Nonrandom allocation of FFV policy. Higher BMI in FFV schools | (1) Models estimate in 3 steps and presented alongside each other (crude, adjusted, +pre-intervention adjusted) to check direction and magnitude of confounding | Described in main text |
|  |  |  | Post-hoc analysis in elementary only schools (NFFV) comparing schools that offered paid FV subscriptions versus those that didn’t | Described in main text and supporting information |
|  |  | Away from null.  Insufficient control for socio-economic circumstances (parental education) | Re-ran analyses adjusting for education in greater resolution (as a 3-level categorical variable rather than binary) | Findings unaltered. See Table A in S6 Text |
|  |  | Away from null  Insufficient control for geographical differences between schools exposed to policy and those not. | Re-run analysis using county (14 levels) as random effect. The main models used region as a fixed effect (4 levels) | Findings unaltered. Results available on request |
| Causal inference (consistency/ triangulation) | Dose response relationship | If FFV is causal then we would expect the CIs to overlap in a way that is either (a) consistent with a dose-response relationship or (b) Shows no heterogeneity between cohorts (if there is no benefit or unintended consequence from being exposed to policy for >1y) | Cohorts have different duration of exposure. Analysis stratified by cohort with formal test of interaction | Described in main text |
| Causal inference (triangulation) | Exposure classification. | Towards null  If taking FFV is causal then we’d expect any effect to be diluted in our main analysis since not all children take the FFV and some NFFV schools offered parents paid subscriptions. | Re-ran analyses after removing the NFFV schools that signed up for parental subscription to FV.  If point estimates are stronger then it lends some support to any findings of an effect. | Described in main text and supporting information |

BMI: body mass index; CI: confidence intervals; FV: fruit and vegetables; FFV: free fruit and vegetables; NFFV: no free fruit and vegetables; MNAR: Missing not at random; MAR: Missing at random
