# Supplementary material for: A nationwide school fruit and vegetable policy and childhood and adolescent overweight: A quasi-natural experimental study
Source: PLoS Med. 2022 Jan 18;19(1):e1003881. doi: 10.1371/journal.pmed.1003881 (PMC8765663; doi:10.1371/journal.pmed.1003881)
Supplement: S1 Text — (DOCX) [file pmed.1003881.s016.docx]

# S1 Text

# Supporting information - Standardizing body mass index

**S1 Text. Standardizing the BMI outcome (BMI standard deviation scores).**

Growth is a continuous non-linear process. To linearize the functional form of the trajectories over the pre- and post-intervention period and thus meet the assumption for the comparison shown in Fig A in S3 Text, BMI was rescaled to internally derived age and sex standardized standard deviation scores (BMI_SDS_). This was done using an approach similar to that outlined by Royston (1995) [1]. Briefly, a power for the transformation of BMI that best normalizes the outcome was selected using a Box-Cox procedure. A multilevel model was then fitted using a 2^nd^ order polynomial function of age with random intercepts and slopes for each term of the age function. The polynomial function was selected from a family of second-degree polynomials, and the function containing the powers with the lowest deviance was used. The estimated variance of BMI from the model, which is a changing function of age, was then used to convert BMI (kg/m^2^) to standard deviation scores. The models were a good fit- the distributions of the BMI_SDS_ were approximately normal conditional on age, and shared similar properties to a standard normal distribution.

Table A shows that this transformation linearized the pre-intervention slopes whereas modelling on the raw scale or using an external growth reference (e.g.; the World Health Organization’s growth reference) to standardize to z-scores did not remove all of the non-linearity. Modelling on this internally standardized scale also improved the fit of the models used to estimate the policy effect.

Table A. Wald tests (p-values) of a non-linear term (quadratic) added to the functional form to model the shape of BMI from 2 to 5.5 years using three different scales, in each of the longitudinal cohorts and with cohorts pooled.

|  |  | BMI (kg/m^2^) | Externally standardized BMI z-scores (WHO) [2] | Internally standardized BMI_SDS_ |
| --- | --- | --- | --- | --- |
| Boys | 2010 | <0.001 | 0.015 | 0.77 |
|  | 2015 | <0.001 | 0.395 | 0.24 |
|  | 2017 | 0.052 | 0.003 | 0.17 |
|  | Pooled | <0.001 | 0.001 | 0.99 |
| Girls | 2010 | 0.009 | 0.064 | 0.28 |
|  | 2015 | <0.001 | 0.83 | 0.526 |
|  | 2017 | 0.398 | 0.038 | 0.065 |
|  | Pooled | <0.001 | 0.055 | 0.272 |

BMI: Body mass index; BMI_SDS_: body mass index standard deviation scores; WHO: World Health Organization.

**References**

1. Royston P. Calculation of unconditional and conditional reference intervals for foetal size and growth from longitudinal measurements. Stat Med. 1995;14(13):1417-36. Epub 1995/07/15. doi: 10.1002/sim.4780141303. PubMed PMID: 7481181.
2. WHO Growth, Reference, Study, Group. WHO Child Growth Standards: Length/height-for-age, weight-for-age, weight-for-length, weight-for-height and body mass index-for-age: Methods and development. Geneva: World Health Organization; 2006.
