# Supplementary material for: A nationwide school fruit and vegetable policy and childhood and adolescent overweight: A quasi-natural experimental study
Source: PLoS Med. 2022 Jan 18;19(1):e1003881. doi: 10.1371/journal.pmed.1003881 (PMC8765663; doi:10.1371/journal.pmed.1003881)
Supplement: S2 Text — (DOCX) [file pmed.1003881.s017.docx]

# S2 Text

# Supporting information - Exposure to the free fruit and vegetable policy classification

**S2 Text. Exposure to FFV policy classification.**

In assigning a duration of exposure to the FFV policy for each cohort, we assume that the elementary school attended at third grade recruitment is the same as attended during first or second grades or that the child had attended a school with the same obligation to the FFV policy. To see how strong this assumption is, we use information from the consent form of the 2017 cohort to give an indication of the number of elementary schools attended and how many were of the same FFV classification. This is not entirely comparable since the consent form in the 2017 cohort, which was administered when the children were in lower secondary school, only asked parents to list up to four elementary schools attended, no dates were attached so children may have attended these schools outside the first to third grade exposure period. Nonetheless, it gives an upper bound estimate of the amount of movement between FFV and NFFV schools and potential for misclassification.

A total of 164 (10%) of children had attended more than one elementary school, of these 69 (42%) had attended both an elementary-only school (NFFV school) and a combined school (FFV) (see Table A and B below). Thus approximately 4% of the sample were exposed to both FFV and NFFV schools. This information was only available in the 2017 cohort.

Table A. Frequency of children by number of elementary schools reported attended from the 2017 cohort.

| Number of schools attended | N (%) |
| --- | --- |
| 1 | 1426 (89.7) |
| 2 | 150 (9.4) |
| 3 | 11 (0.7) |
| 4 | 3 (0.2) |

Table B. History of attendance (exposure) at FFV and NFFV schools in children that attended more than one school (n = 164) from the 2017 cohort.

| Exposure history | N (%) |
| --- | --- |
| FFV only | 13 (8) |
| NFFV only | 82 (50) |
| FFV and NFFV | 69 (42) |
| Total | 164 |

FFV: free fruit and vegetables; NFFV: no free fruit and vegetables (controls).
