# Supplementary material for: A nationwide school fruit and vegetable policy and childhood and adolescent overweight: A quasi-natural experimental study
Source: PLoS Med. 2022 Jan 18;19(1):e1003881. doi: 10.1371/journal.pmed.1003881 (PMC8765663; doi:10.1371/journal.pmed.1003881)
Supplement: S3 Text — (DOCX) [file pmed.1003881.s018.docx]

# S3 Text

# Supporting information - Further information about estimating the policy effect

**S3 Text. Longitudinal estimation of the FFV policy effect.**

Fig A below illustrates how the FFV policy effect at grade 3 (~8.5 years) was estimated using the longitudinal datasets (cohorts 2010, 2015, and 2017 for the BMI_SDS_ and OW/OB outcomes). The pre- and post-FFV policy trajectories were modelled using linear splines with a knot prior to the policy exposure at 5.5 years (see eq. (1) in main text). The splines are formulated as follows with age centered (c_age) at the pre-policy knot (c_age = age – 5.5y):

$S_{1}=\left\{ \begin{aligned} c\_age \\ 0 \end{aligned} \begin{matrix} c\_age\leq0 \\ c\_age>0 \end{matrix} \right.$

$S_{2}=\left\{ \begin{aligned} 0 \\ c\_age \end{aligned} \begin{matrix} c\_age\leq0 \\ c\_age>0 \end{matrix} \right.$

(eq. s1)

The grey area in Fig A represents the ages when children were introduced to FFV which was in first grade of school. To avoid bias in the estimate of the FFV post-intervention slope caused by including datapoints at ages when some children in FFV schools were exposed and some were not, all data around this period were removed leaving only third grade as the post-intervention measures for the analysis of outcomes at ~8 year, and third and eight grade measures for the analysis of outcomes at ~13 years. Removal of observations around the introduction of the intervention also prevents future measures from influencing the estimation of the pre-intervention slope which makes no sense causally and would introduce bias to the estimate of the policy effect. S2 Fig shows the individual data included in the analyses.

To linearize the functional form of the relationship for the BMI outcome which has a complex shape early in life, we also derived internally standardized BMI standard deviation scores (BMI_SDS_) – see S1 Text.

Estimating the FFV policy effect at age 13 in the 2017 cohort

To estimate the longer-term effect of the FFV policy at age 13 years in the 2017 cohort, the linear spline model illustrated in Fig A and equation (1) of the main text was extended to incorporate an extra knot in third grade (age 8.5 years). The model was simplified to constrain groups to have the same pre-intervention slope. As shown in Fig 1 (main text) and S4 Fig, and in S3 and S4 Table, this was a reasonable assumption. The basic model can thus be written as:

$E\left( Y \right)=\beta_{0}+\beta_{1}S_{1}+\beta_{2}S_{2}+\beta_{3}S_{3}+\gamma_{0}I+\gamma_{2}I*S_{2}+\gamma_{3}I*S_{3}$

(eq. s2)

where *I* is a binary variable indicating FFV exposure, and $S_{1}$, $S_{2}$ and $S_{3}$ are linear splines of *age* (years) centered at the pre-policy age (*c_age*=*age* - 5.5) with knots at age 5.5 years (pre-policy) and 8.5 years (grade 3) as follows:

$S_{1}=\left\{ \begin{aligned} c\_age \\ 0 \end{aligned} \begin{matrix} c\_age\leq0 \\ c\_age>0 \end{matrix} \right.$

$$S_{2}=\left\{ \begin{aligned} 0 c_{age}\leq0 \\ c_{age}-3 0<c_{age}\leq3 \\ 3 c_{age}>3 \end{aligned} \right.$$

$$S_{3}=\left\{ \begin{aligned} 0 \\ c\_age \end{aligned} \begin{matrix} c\_age\leq3 \\ c\_age>3 \end{matrix} \right.$$

(eq. s3)

$\beta_{0}, \beta_{1}, \beta_{2}$ and $\beta_{3}$ describe the outcome ($E\left( Y \right)$) at 5.5 years and the pre- ($\beta_{1})$and post-intervention slopes ${(\beta}_{2}, \beta_{3})$respectively in the control (NFFV) group. $\gamma_{0}, \gamma_{2}$ and $\gamma_{3}$ are the mean difference in intercept at 5.5 years and mean difference in post-intervention slopes between groups.

The counterfactual is the trajectory that the FFV group would have taken in absence of the intervention and is estimated by the post intervention slopes in the NFFV group. The difference between the FFV group and the counterfactual at age 13 years is thus an estimate of the policy effect at age 13 years and is given by ${(\gamma}_{2}*3+\gamma_{3}*4.5)$.


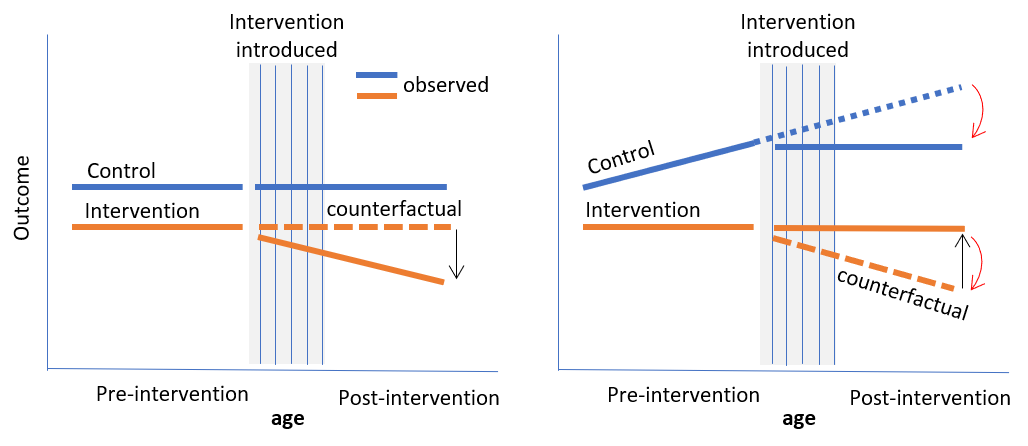


Fig A. Illustration of the estimation of the free fruit and vegetable policy effect in the longitudinal analysis.

The counterfactual is that the slope in the intervention group would have changed the same way post-intervention as observed in the control group. This is shown by the dashed line and indicated by the red arrows. The effect is thus estimated by the difference between what was observed in the intervention group versus the counterfactual, as represented by the black arrow. The figure on the left shows a credible comparison for such a policy effect estimator where the pre-intervention slopes are similar. The situation on the right is less credible - the pre-intervention slopes and intercepts are quite different between groups, in this setting the counterfactual is less believable.
