# Supplementary material for: A nationwide school fruit and vegetable policy and childhood and adolescent overweight: A quasi-natural experimental study
Source: PLoS Med. 2022 Jan 18;19(1):e1003881. doi: 10.1371/journal.pmed.1003881 (PMC8765663; doi:10.1371/journal.pmed.1003881)
Supplement: S4 Text — (DOCX) [file pmed.1003881.s019.docx]

# S4 Text

# Supporting information - Additional information on the allocation of the free fruit and vegetable policy

**S4 Text. Regional patterning of combined elementary and secondary (FFV) and elementary-only schools (NFFV).**

Table A shows the national distribution of combined elementary and secondary schools (those obligated by the free school fruit and vegetable policy) by region. The Northern region has the highest proportion of combined schools (FFV) and the Southern/Eastern region the lowest. This pattern was mirrored in our data sets (see S1 and S2 Tables).

Table A. Mean national distribution of combined (FFV) and pure elementary (NFFV) schools from 2010, 2012, and 2015, by region and total.

|  | Total | Elementary/NFFV schools  (1-7^th^ grade) | | Combined/FFV schools  (1-10^th^ grade) | |
| --- | --- | --- | --- | --- | --- |
| Region | N | n | % | n | % |
| Northern | 392 | 167 | 42.6 | 225 | 57.4 |
| Southern/Eastern | 1090 | 835 | 76.6 | 255 | 23.4 |
| Western | 565 | 414 | 73.2 | 151 | 26.8 |
| Central | 420 | 291 | 69.4 | 129 | 30.6 |
| Total | 2467 | 1706 | 69.2 | 760 | 30.8 |

Source: The Norwegian Directorate of Education. 
FFV: free fruit and vegetables; NFFV: no free fruit and vegetables (controls).
