# Supplementary material for: A nationwide school fruit and vegetable policy and childhood and adolescent overweight: A quasi-natural experimental study
Source: PLoS Med. 2022 Jan 18;19(1):e1003881. doi: 10.1371/journal.pmed.1003881 (PMC8765663; doi:10.1371/journal.pmed.1003881)
Supplement: S5 Text — (DOCX) [file pmed.1003881.s020.docx]

# S5 Text

# Supporting information - Directed Acyclic Graph

**S5 Text. Directed Acyclic Graph.**

The allocation of FFV policy cannot be considered “as if” random due to differences in the distribution of combined (FFV) schools between regions. This is likely to be driven in part by differences in the population density between regions. Northern regions of Norway are more rural and combined (FFV) schools are more likely to be located in regions of lower population density – as reflected in our data (see S2 Table). A Directed Acyclic Graph (DAG) was thus constructed to determine what variables to adjust for to obtain a causal estimate of the FFV policy effect (Fig A below).

The DAG in Fig A has an arrow from FFV school to obesity-related outcomes acting through the consumption of the fruit and vegetables and through diet. Region and population density have direct links to FFV allocation and are a sufficient adjustment set to estimate the causal effect of the FFV policy.

Cohort determines the duration of exposure to the FFV policy and may also affect intake of the fruit and vegetables and any secular dietary changes. One might therefore expect a different policy effect between cohorts. Similarly, there may be differences in response to the policy between boys and girls and between children of different socio-economic background (of which parental education is a marker), as indicated in the DAG. For these reasons, we also present results stratified on these factors.

A key assumption encoded in the DAG is that parental education is independent of FFV allocation conditional on region and population density. Table A shows that this assumption did not hold - in several region and population density strata there was a higher proportion of children from parents with a higher education attending elementary only (NFFV) schools, hence, education was additionally adjusted for. Pre-intervention BMI, which is not included in the DAG, may also capture other differences in the child’s obesogenic environment linked to region, population density, and socio-economic factors, and so was adjusted for in an additional model.


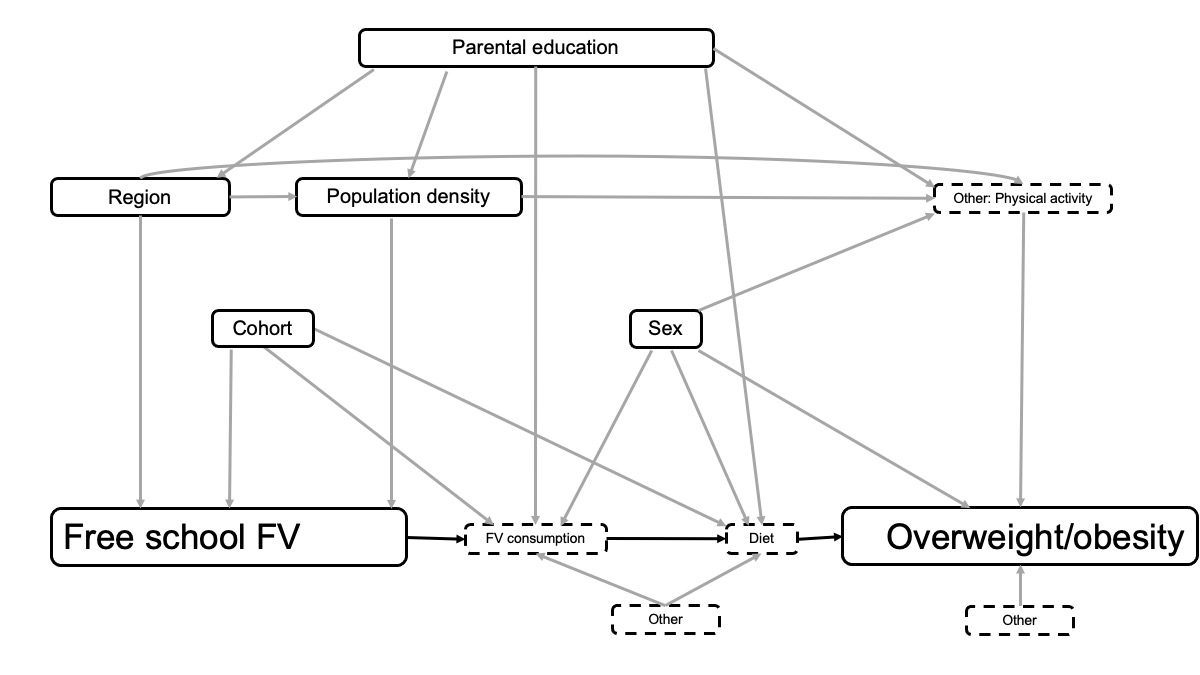


Fig A. Directed Acyclic Graph encoding the hypothesized causal relations between FFV allocation and weight status outcomes.

Dashed indicated unmeasured variables. FFV: free fruit and vegetables; FV: fruit and vegetables.

Table A. Association between FFV and education conditional (stratified) on region and population density (pooled across all cohorts).

| Region | Population density | Group | <Higher education | Higher+ education | p^†^ |
| --- | --- | --- | --- | --- | --- |
| Southern / Eastern | Urban (> 50000) | NFFV | 1818 (40.0%) | 2845 (61.0%) |  |
|  |  | FFV | 334 (50.2%) | 332 (49.9%) | <0.001 |
|  | Semi-urban (15-50000) | NFFV | 115 (44.6%) | 143 (55.4%) |  |
|  |  | FFV | 5 (100%) | 0 | 0.014 |
|  | Rural (< 15000) | NFFV | 10 (45.5%) | 12 (54.6%) |  |
|  |  | FFV | 21 (58.3%) | 15 (41.7%) | 0.34 |
|  |  |  |  |  |  |
| Western | Urban (> 50000) | NFFV | 743 (42.5%) | 1005 (57.5%) |  |
|  |  | FFV | 163 (43.1%) | 215 (56.9) | 0.83 |
|  | Semi-urban (15-50000) | NFFV | 34 (50.8%) | 33 (49.3%) |  |
|  |  | FFV | 40 (66.7%) | 20 (33.3%) | 0.069 |
|  | Rural (< 15000) | NFFV | 91 (50.3%) | 90 (49.7%) |  |
|  |  | FFV | 71 (47.7%) | 78 (52.4%) | 0.64 |
|  |  |  |  |  |  |
| Central | Urban (> 50000) | NFFV | 138 (25.8%) | 396 (74.1%) |  |
|  |  | FFV | 81 (32.5%) | 168 (67.5%) | 0.052 |
|  | Semi-urban (15-50000) | NFFV | 97 (44.5%) | 121 (55.5%) |  |
|  |  | FFV | 39 (36.5%) | 68 (63.5%) | 0.17 |
|  | Rural (< 15000) | NFFV | 148 (55.9%) | 117 (44.2%) |  |
|  |  | FFV | 45 (61.6%) | 28 (38.4%) | 0.38 |
|  |  |  |  |  |  |
| Northern | Urban (> 50000) | NFFV | 16 (28.6%) | 40 (71.4%) |  |
|  |  | FFV | 69 (66.4%) | 35 (33.7%) | <0.001 |
|  | Semi-urban (15-50000) | NFFV | 294 (46.2%) | 343 (53.9%) |  |
|  |  | FFV | 109 (34.1%) | 211 (65.9%) | <0.001 |
|  | Rural (< 15000) | NFFV | 62 (48.1%) | 67 (51.9%) |  |
|  |  | FFV | 94 (61.0%) | 60 (39.0%) | 0.029 |

^†^: Chi-squared test of association in each region-population density stratum.

FFV: free fruit and vegetables; NFFV: no free fruit and vegetables (controls).
