# Supplementary material for: A nationwide school fruit and vegetable policy and childhood and adolescent overweight: A quasi-natural experimental study
Source: PLoS Med. 2022 Jan 18;19(1):e1003881. doi: 10.1371/journal.pmed.1003881 (PMC8765663; doi:10.1371/journal.pmed.1003881)
Supplement: S6 Text — (DOCX) [file pmed.1003881.s021.docx]

# S6 Text

# Supporting information - Secondary/supplementary analyses

## Using different classifications of education to control for socioeconomic differences

**S6 Text. Sensitivity analysis with different classifications of parental education.**

In the main analyses, differences in parental education between groups exposed to the FFV policy were adjusted for using a dichotomous indicator of highest parental education (higher education in university/college or high school or less). As a response to the review process, a sensitivity analysis to check the robustness of our results to this choice, and as a secondary analysis to gain an insight into the potential direction and magnitude of residual confounding caused by inadequate adjustment for socio-economic differences between groups we repeated the analyses in the most adjusted pooled-cohort models for each outcome after controlling for parental education in two further ways. First education was entered as a 3-level variable (intermediate or less, undergraduate, postgraduate). Second education was kept in its full 9-level form corresponding with codes from The International Standard Classification of Education, ISCED (no education, primary, lower secondary, upper secondary basic, upper secondary final year, post-secondary non-tertiary, undergraduate, graduate, and postgraduate) and entered as an ordinal variable with the assumption of linearity. Low cell counts make it problematic to further subdivide education and adjust for it as a categorical variable. Table A shows the results of these analyses alongside the original results from the main analyses. The way in which parental education was derived had negligible effect on the policy-effect estimates.

Table A. Estimates of the FFV policy effect* on outcomes (BMI_SDS_, OW/OB, WC, and WtHR) in boys and girls at 8.5 years when adjusting for parental education using different classifications.

|  | Models adjusting for education as 2-level variable^(a)^ | | Models adjusting for education as 3-level variable^(b)^ | | Models adjusting for education as 9-level ordinal variable^(c)^ | |
| --- | --- | --- | --- | --- | --- | --- |
|  | Policy effect estimate (95% CI)† | p | Policy effect estimate (95% CI)† | p | Policy effect estimate  (95% CI)† | p |
| Boys |  |  |  |  |  |  |
| BMI_SDS_ | 0.05 (-0.04, 0.14) | 0.29 | 0.05 (-0.04, 0.14) | 0.29 | 0.05 (-0.04, 0.13) | 0.31 |
| OR of OW/OB | 1.20 (0.86, 1.66) | 0.28 | 1.20 (0.87, 1.66) | 0.27 | 1.19 (0.86, 1.64) | 0.29 |
| WC (cm) | 0.3 (-0.3, 0.8) | 0.30 | 0.3 (-0.3, 0.8) | 0.30 | 0.3 (-0.3, 0.8) | 0.31 |
| WtHR | 0.002 (-0.002, 0.006) | 0.28 | 0.002 (-0.002, 0.006) | 0.29 | 0.002 (-0.002, 0.006) | 0.29 |
| Girls |  |  |  |  |  |  |
| BMI_SDS_ | 0.04 (-0.04, 0.13) | 0.33 | 0.04 (-0.05, 0.12) | 0.38 | 0.04 (-0.05, 0.12) | 0.39 |
| OR of OW/OB | 1.03 (0.75, 1.39) | 0.87 | 1.02 (0.75, 1.38) | 0.92 | 1.00 (0.74, 1.36) | 0.98 |
| WC (cm) | 0.04 (-0.6, 0.65) | 0.90 | 0.03 (-0.6, 0.6) | 0.92 | 0.02 (-0.59, 0.63) | 0.95 |
| WtHR | -0.000 (-0.004, 0.004) | 0.98 | -0.000 (-0.004, 0.004) | 0.96 | -0.000 (-0.004, 0.004) | 0.91 |

*Results are from the most adjusted models as presented in the main text. BMI_SDS_ and OW/OB models include cohort, region, population density, highest parental education (as described below), and BMI_SDS_ prior to the intervention. WC and WtHR models include cohort, region, population density, and highest parental education (as described below).  
†Expressed as the difference in outcome for BMI_SDS_, WC, and WtHR or odds ratio (OR) for OW/OB versus the counterfactual (as estimated using the NFFV schools) with 95% CI. 
^(a)^ Parental education split into two categories as in the main analyses (<higher education, higher education).
^(b)^ Parental education split into three categories (intermediate or less, undergraduate higher education, postgraduate higher education).
^(c)^ Parental education treated as a 9-level ordinal variable (no education, primary, lower secondary, upper secondary basic, upper secondary final year, post-secondary non-tertiary, undergraduate, graduate, and postgraduate).
BMI_SDS_: body mass index standard deviation scores; CI: confidence interval; FFV: free fruit and vegetables; NFFV: no free fruit and vegetables; OR: odds ratio; OW/OB: overweight and obesity; WC: waist circumference in centimeters; WtHR: waist to heigh ratio.
