# Supplementary material for: A nationwide school fruit and vegetable policy and childhood and adolescent overweight: A quasi-natural experimental study
Source: PLoS Med. 2022 Jan 18;19(1):e1003881. doi: 10.1371/journal.pmed.1003881 (PMC8765663; doi:10.1371/journal.pmed.1003881)
Supplement: S7 Text — (DOCX) [file pmed.1003881.s022.docx]

# S7 Text

# Supporting information - Threats to the validity of the study

**S7 Text: National policy initiatives and co-interventions occurring over the time frame of the study.**

Table A lists national level policies and co-interventions that may have altered outcomes assessed in our study. It also includes, to the best of our knowledge, any known pilot studies that stemmed from national initiatives.

While there have been several national campaigns and policies that if effective, would have acted on the weight outcomes in our study, many occurred post-2014 and so would have only potentially influenced outcomes at age 13 in the 2017 cohort, and all would have reduced the obesogenic environment across all schools regardless of exposure to the free school fruit and vegetable policy. It seems unlikely that these co-interventions would have biased our comparisons between schools to an extent that would alter our conclusions. Therefore, the risk of bias from these co-interventions is assumed to be low.

Table A. National interventions and policies that were introduced during the time frame of our current study (2007 to 2017) and which may have affected weight outcomes.

| **Time varying confounding factors** | **Population-level policies and interventions between 2007-2017** |
| --- | --- |
| Physical activity | Action plan on physical activity 2005-2009: included a goal to increase proportion of children and adolescents who are in moderate physical activity for 60 minutes each day.  2014-2015: Pilot to increase hours of physical activity in secondary schools (it is assumed the target population was grades 8 to 10). A total of 7 schools started in 2016, while 30 additional schools were included in 2017. |
| Increase healthy eating | Action plan on nutrition 2007-2011: Action plan on nutrition aiming to improve nutrition in the population towards the recommendations from health authorities by (relevant measures): focus on healthy meals in school by school meal guidelines; fruit and vegetables in school; limit access to soda in schools; limit marketing of unhealthy food and beverages to children; several pilots throughout the country with various models for offering school meals (breakfast and/or lunch); increase knowledge and skills about food, food preparation, nutrition and health in elementary schools.  2007-2013: Voluntary cooperation with the industry to restricting marketing of unhealthy food/beverages to children under the age of 13. Started in 2007, was re-enforced in 2013.  2011-date: Thirteen food-based advice from health authorities to the population, one is eat five-a-day (of fruit and vegetables).  2014: Establishment of National Centre for food, health and physical activity aimed to strengthen each kindergarten and school’s role as a health promoting and preventive arena.  2014-2015: Point out the need for 20 min eating break; and strengthen practical cooking skills and facilitate healthy food and meals in schools and kindergarten.  2015-date: Matjungelen: initiative to make children into *agents of change* focusing on healthy and sustainable dietary choices.  2015: Revised national guidelines “food and meals in school”: Specifies that all elementary and secondary schools should offer fruit and vegetables to pupils each day; baked goods and foods with high amounts of sugar/fats should only be used on special occasions; candy, chocolate, chips, and other snack should be limited and not provided in schools.  Action plan on nutrition 2017-2021: Aiming to improve diet according to nutritional recommendations. Relevant measures in the plan: Monitor work restricting marketing of unhealthy food and beverages to children; promote healthy meals in schools; focus on the need for a 20-minute lunch break; mobilize children as agents of change (Matjungelen) focusing on healthy and sustainable dietary choices; increase practical skills; and increase knowledge and resources to teachers in health economics. |
