# Supplementary material for: A nationwide school fruit and vegetable policy and childhood and adolescent overweight: A quasi-natural experimental study
Source: PLoS Med. 2022 Jan 18;19(1):e1003881. doi: 10.1371/journal.pmed.1003881 (PMC8765663; doi:10.1371/journal.pmed.1003881)
Supplement: S8 Text — (DOCX) [file pmed.1003881.s023.docx]

# S8 Text

# Supporting information - Threats to the validity of the study

## Risk of bias in non-randomized comparisons

**S8 Text. ROBINS-I tool for risk of bias in non-randomized comparisons.**

To help understand the potential risk of bias in our study and provide a framework for considering bias, two of the authors (BØ, AKW) completed, discussed and agreed on the ROBINS-I tool [1]. Fig A summarizes this assessment in our study. Risk of bias due to confounding (D1) is an obvious concern since the intervention cannot be considered “as if” random, but the use of a Directed Acyclic Graph (DAG) to determine a sufficient adjustment set to obtain a causal estimate, and the ability to adjust for pre-intervention differences in the outcome and triangulate the evidence with additional comparisons to check for residual confounding and inform the conclusions would suggest that while risk of confounding is moderate, the ability to adjust for further confounders or for more accurate indicators of potential confounders is unlikely to alter the magnitude of the estimates to an extent that it would materially alter the conclusions of our study. Risk of bias due to deviations from intended interventions (D4) is likely since our classification does not account for children who changed school. However, as stated, we predict that less than 4% of the sample would have attended both a FFV and NFFV school and so bias from this domain is also unlikely to be sufficient in magnitude to alter our conclusions. Other domains of bias assessed by ROBINS-I were considered low and the overall assessment was considered moderate since the study is not comparable to a well-performed randomized controlled trial.


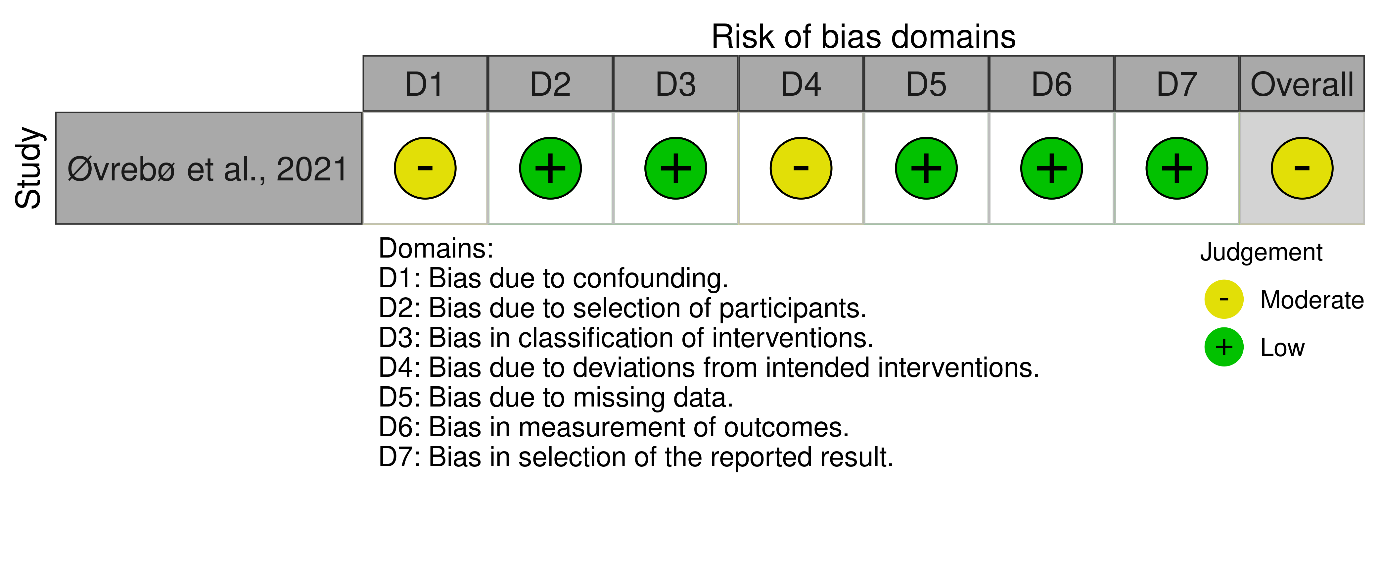


Fig A. Visualization of the ROBINS-I risk of bias for non-randomized interventions assessment of our study [2].

**References**

1. Sterne JA, Hernan MA, Reeves BC, Savovic J, Berkman ND, Viswanathan M, et al. ROBINS-I: a tool for assessing risk of bias in non-randomised studies of interventions. BMJ. 2016;355:i4919. Epub 2016/10/14. doi: 10.1136/bmj.i4919. PubMed PMID: 27733354; PubMed Central PMCID: PMCPMC5062054
2. McGuinness, LA, Higgins, JPT. Risk-of-bias VISualization (robvis): An R package and Shiny web app for visualizing risk-of-bias assessments. Res Syn Meth. 2020; 1- 7. https://doi.org/10.1002/jrsm.1411
