# Supplementary material for: A nationwide school fruit and vegetable policy and childhood and adolescent overweight: A quasi-natural experimental study
Source: PLoS Med. 2022 Jan 18;19(1):e1003881. doi: 10.1371/journal.pmed.1003881 (PMC8765663; doi:10.1371/journal.pmed.1003881)
Supplement: Attachment — Submitted filename: Response to reviewers.docx [file pmed.1003881.s024.docx]

**Requests from the editors:**

- Please revise your title according to PLOS Medicine's style. Your title must be nondeclarative. It should begin with main concept if possible. "Effect of" should be used only if causality can be inferred, i.e., for an RCT. Please place the study design in the subtitle (ie, after a colon). See PLOS Medicine's website for examples: <https://journals.plos.org/plosmedicine/>

*Answer: We have changed the title in accordance with PLOS Medicine’s style.*

- Please update your Data Availability Statement to remove ‘for non-Norwegians’, as the URL provided appears to contain information aimed at both those that do and those that do not hold a Norwegian electronic ID (if I have understood correctly).

*Answer: We have updated this Statement.*

- In the Abstract Methods and Findings please include the important dependent variables that are adjusted for in the main analyses.

*Answer: We have made the changes in the abstract, see page 2, lines 43-45.*

- Please begin the last sentence of the Abstract Methods and Findings section: ‘The main limitations of this study are…’.

*Answer: We have made the changes in Abstract Methods and Findings, see page 3, lines 60-62.*

- In your Abstract Conclusions, please address the study implications without overreaching what can be concluded from the data; the phrase "In this study, we observed ..." may be useful, given that the study has an observational design.

*Answer: We have made the changes in Abstract Conclusions, see page 3, lines 56-58.*

- At this stage, we ask that you include a short, non-technical Author Summary of your research to make findings accessible to a wide audience that includes both scientists and non-scientists. The Author Summary should immediately follow the Abstract in your revised manuscript. This text is subject to editorial change and should be distinct from the scientific abstract. Please see our author guidelines for more information: <https://journals.plos.org/plosmedicine/s/revising-your-manuscript#loc-author-summary>

*Answer: We have written Author Summary in the revised manuscript. See page 3-4, lines 64-89.*

- Please confirm that informed consent (provided by parents/guardians) was written (rather than oral) for all of the involved cohorts (the current manuscript suggests this was the case but it would be useful to have a short sentence confirming).

*Answer: We have included the following sentence: “Detailed information about the studies (NCGS and NYGS) were sent to parents or guardians prior to each survey, and the School Health Service obtained written informed consent from parents or other legal guardians on behalf of the Norwegian Institute of Public Health.” Page 14, lines 338-341.*

- Thank you for including the completed STROBE checklist as Supporting Information. Please add the following statement, or similar, to the Methods: "This study is reported as per the Strengthening the Reporting of Observational Studies in Epidemiology (STROBE) guideline (S1 Checklist)."

*Answer: We have included this sentence in the Method section, please see page 7, lines 146-147.*

- Please update the STROBE checklist to use section and paragraph numbers, rather than page numbers.

*Answer: We have also updated the checklist indication section and paragraph numbers when appropriate. Please see the new uploaded STROBE checklist in supporting information (S1 Checklist).*

- Did your study have a prospective protocol or analysis plan? Please state this (either way) early in the Methods section.

a) If a prospective analysis plan (from your funding proposal, IRB or other ethics committee submission, study protocol, or other planning document written before analyzing the data) was used in designing the study, please include the relevant prospectively written document with your revised manuscript as a Supporting Information file to be published alongside your study, and cite it in the Methods section. A legend for this file should be included at the end of your manuscript.

b) If no such document exists, please make sure that the Methods section transparently describes when analyses were planned, and when/why any data-driven changes to analyses took place.

c) In either case, changes in the analysis-- including those made in response to peer review comments-- should be identified as such in the Methods section of the paper, with rationale.

*Answer: Our analysis strategy was formulated prior to conducting the analyses, this included outcomes, modelling approach to estimate the intervention effect, main comparisons and adjustments, explorative analyses to inform the parameterization of the models, and a list of sensitivity analyses. We had planned to expand this to a formal document for pre-registration, unfortunately however, delays and uncertainties with data access combined with the impact of the COVID pandemic and time constraints from the grant funding this work prevented this. The following text is included in the manuscript: “The design was driven by the policy rollout and the availability of datasets from the Norwegian Growth Cohort. The analysis strategy was planned a priori but we did not pre-register a protocol due to a combination of delays in data access and fall out from the COVID pandemic. Any secondary or post-hoc analyses that were done in response to the results or from the review process are defined in the text and in supporting information” (page 5, lines 141-145). We have also described supplemental and sensitivity analysis (including those made in response to peer review) under methods supplemental and sensitivity analysis, page 14, lines 321-331.*

- Please remove the Authors’ contributions, Data availability, Funding, and Conflicts of Interest sections from the end of the main text. In the event of publication, this information will be published as metadata based on your responses to the relevant questions in the submission form.

*Answer: We have removed these paragraphs in the main text.*

- Please relocate your Ethics approval section from the end of the main text to the Methods.

*Answer: We have relocated the ethics section, see page 14, lines 334-341.*

**Comments from the reviewers:**

**Reviewer #1**: The authors estimate the impact of a policy to provide free fruit and vegetables (FFV) at school meals in Norway on outcomes related to adiposity and weight distribution.  FFV programs were nonrandomly allocated and for causal inference the authors use longitudinal observational methods with correction for some potential confounding variables.  These methods do not detect an impact of FFV policy exposure on BMI, odds of OW/OB, or waist circumference.  I feel strongly that it is important to publish null findings, so I have no concerns about that.  I am only superficially familiar with the methods used in this analysis and so will rely on the statistical reviewer for a more detailed review in that regard.

1) My main constructive critique is that the analysis appears not to exclude the possibility of meaningful effects on the odds of OB/OW, especially in the cohort with longest exposure where the estimate appears imprecise.  In general, the estimates do not appear precise enough to exclude meaningful effects.  I think this is something that could be mostly addressed via revision of the text.  It is rarely possible to demonstrate a true null; what is possible is to exclude effect sizes of a certain magnitude.  I think the paper should acknowledge that the analysis does not exclude potentially meaningful effect sizes and is not necessarily a failure to replicate previous positive findings (in the sense that 95% CIs overlap considerably), and address this point quantitatively.  What size effects can be reasonably excluded by this analysis, and what size effects cannot be excluded?  Discussion of this, and the uncertainty of interpretation it implies, would be helpful.

*Answer: Thank you for this very helpful comment. As this comment relates to some of the reviewers’ other comments, we refer to our answers to those comments to provide an extensive answer and to minimize duplication of answers.*

*We agree that our study does not rule out the possibility of a meaningful effect on the odds of OW/OB, however, overall we conclude that the FFV policy is unlikely to have a materially significant effect on the weight outcomes (additional details in answer to comment #2 and #4). We have tried to consistently report that we found little evidence of a beneficial or unintended effect from the policy, which is not the same as no effect (or null findings). As the reviewers says, “it is rarely possible to demonstrate a true null”. We have gone through the manuscript and edited the text to make sure this is consistently written. Additionally, where we have contrasted our results with the study from Arkansas US (Qian et al. 2016^[1]^), we have modified the text, noting that our pooled results fall within the plausible bounds of their reported findings (page 21, lines 522-523). Our comment regarding effect sizes and the Arkansas’ study is elaborated further in answer to comment #4.*

2) My second constructive critique is that I am not as convinced as the authors that the conclusions are at a low risk of being impacted by bias, as reflected in the abstract: "Residual confounding and exposure misclassification are the main threats to the validity of our estimates but are unlikely to be of sufficient magnitude to affect our conclusions".  Measurement and categorization of potential confounding variables tends to be crude (e.g., dichotomizing educational status), and the number of potential confounding variables included is limited (e.g., economic status is not directly considered).  I'm not knowledgeable enough about the methods used in this study to form a strong opinion on this, but based on the information presented and my general familiarity with this class of methods, I'll say I'm skeptical of the assertion that the risk of bias is low.  For example, the ORs for the pre-intervention trajectories of OB/OW in table S7 have confidence intervals that include the possibility of substantial differences (e.g., 0.75 to 1.20), suggesting that it is possible that OB/OW risk trajectories in the intervention children were not well matched with controls.  Perhaps the statistical reviewer can contribute an opinion on this, but my opinion is that the assertions of low risk of bias should be removed or softened.

*Answer: We agree with the reviewer that bias is the main concern, as it is in any observational study which includes those that are quasi-experimental such as ours. We did state that the risk of bias in our study is moderate (not low), based on the ROBINS-I tool (see supporting information – S8 Text and S12 Fig, and page 26, lines 628-629 of main text). However, while we think the chance of bias is moderate, we have tried to assess whether any potential bias is of sufficient magnitude and direction to alter our conclusions. To do this we exploited the study design and datasets with a battery of comparisons, checks, and sensitivity and secondary analysis to triangulate the evidence and assess the robustness of the main results. These are detailed in S12 Table in supporting information. The triangulation of our findings, as highlighted in an article by Lawlor et al (2017)^[2]^, led us to conclude that the FFV policy is unlikely to have had a materially significant effect on these weight-related outcomes.*

*Despite this we agree with the reviewer that the language could be softened and have now done this. For example, in the abstract methods and findings: “The main limitations of this study are residual confounding and exposure misclassification but these are probably unlikely to be of sufficient magnitude to alter our conclusions.” (page 3, lines 52-53).*

*The reviewer’s point on potential confounding is dealt with in response to point #8 below.*

More detailed comments, including some related to the above, are below:

3) I see no evidence that the study was preregistered.  This increases risk of bias.  While I am less concerned about this due to the null primary outcome, it does undermine the internal validity of the findings.

*Answer: Our analysis strategy was formulated prior to conducting the analyses. This included the outcomes, the modelling approach to estimate the intervention effect, the main comparisons and adjustments, explorative analyses to help inform the finer detail of the models, and a list of sensitivity analyses. We had planned to expand and formalize this document into a pre-registered protocol but were unable to because of delays and uncertainties with data access, the impact of the COVID pandemic and the grant funding timeline. The following text is now included in the manuscript: “The design was driven by the policy rollout and the availability of datasets from the Norwegian Growth Cohort. The analysis strategy was planned a priori but we did not pre-register a protocol due to a combination of delays in data access and fall out from the COVID pandemic. Any secondary or post-hoc analyses that were done in response to the results or from the review process are defined in the text and in supporting information” (page 6, lines 141-145). We also include the following under study limitations in the discussion (page 26, lines 627-628): “The lack of a pre-registered protocol for our study may undermine findings even though little evidence for a policy effect was observed”.*

*We believe that our analysis strategy is a thorough and transparent attempt to investigate the magnitude and direction of bias. Any post-hoc analyses were done solely as sensitivity analyses to test the robustness of the main findings, and these are identified as post-hoc in the manuscript where this was the case. We include the following text in the methods: “Any secondary or post-hoc analyses that were done in response to the results or from the review process are defined in the text and in supporting information.” (page 6, lines 144-145). We have also described supplemental and sensitivity analysis (including those made in response to peer review) under methods supplemental and sensitivity analysis, page 14, lines 321-331.*

4) The analyses included 6619 to 7810 children, and only a minority of those were in the FFV group (21% in table 1).  How much power did this analysis have to detect significant and quantitatively meaningful effects on BMI and OB/OW?  What is the likelihood that the finding is null because the sample size is too small?  I would expect that if this intervention is effective, the effect size would be quite small.  I wouldn't expect offering F/V to children at school to have a large impact on BMI.  I see that the 95% confidence intervals around the estimated odds ratios for OB/OW are quite wide, for example in figure 2b and especially figure 4b.  This raises the question of whether the underlying data are up to the task of detecting a plausible (presumably small) effect size.

*Answer: The reviewer is correct that approximately 20% of the children were exposed to the FFV policy (about 30% in one cohort), and we agree with the reviewers that free fruit and vegetable provision is unlikely to yield large impacts on BMI.*

*A post-hoc power analysis of observed effect sizes would not provide any additional information over and above the p-value, i.e, power will always be low to detect an observed difference when the p-value is large- power here is just a re-configuration of the p-value. If researchers are interested in being able to detect effect sizes within the plausible range of our estimates in a future study, then our results are useful inputs for future sample size calculations.*

*We agree that the ORs for OW/OB do not exclude potentially important and plausible effect sizes, in particular for the main pooled result for boys. Here, the OR in boys pooling across cohorts was 1.2 (95% CI: 0.86, 1.66) which can be expressed as an absolute difference in prevalence, averaging over other characteristics i.e.; marginal effects, giving a value of +2.5% (95%CI: -0.5, 5.6%) p=0.11. It does not exclude potentially detrimental effects in the other direction. The corresponding association in girls was +0.3% (95%CI: -2.9, 3.7%), which is perhaps more informative.*

*As a general point, we have focused on the confidence intervals for the estimate of the policy effect in our interpretation rather than statistical significance. Across all other outcomes i.e., BMI and waist circumference, the plausible size of the policy-effect based on the confidence intervals is more informative and we think supports the conclusion that there was little evidence for a benefit or unintended consequence. Nonetheless we have now (a) flagged the need for larger studies to detect or rule out small but meaningful effects on OW/OB outcomes (“Studies should also be sufficiently large to detect small but potentially meaningful population level effects on overweight and obesity outcomes”, page 25, lines 604-605); (b) removed the declaration of no strong evidence for benefit or unintended consequence in the title and text; and (c) softened and qualified our discussion and conclusion with respect to this point (e.g.: when comparing our results to the Arkansas US study (page 21-22, lines 519-530) and our Abstract conclusion “In this study we observed little evidence that the Norwegian nationwide FFV policy had any notable beneficial effect or unintended consequence on weight status among Norwegian children and adolescents”).*

5) This is not a critique, just a comment.  The theory of change for this intervention leaves several opportunities for the program to fail to impact OB/OW.  Policy enacted -> F/V provided -> F/V consumed -> F/V displace more energy-dense foods -> total energy intake declines -> lower BMI.  I don't find it surprising that effect size on OB would be small or zero.

*Answer: We agree with the reviewer on this. We still believe our study is important, especially for policy- and decision makers, as school fruit and vegetable policies and programs are relatively common and implemented in the same simple manner as was done in Norway 2007-2014. Under interpretation in the discussion, we comment on the link between provision of fruit and vegetables and compensatory behavior changes and why this might result in no overall aggregated policy effect (page 23, lines 555-562).*

6) It is a significant weakness that the data do not include a measure of the impact of the intervention on F/V consumption.  This isn't strictly required since the objective of the study is to evaluate the policy, but it does complicate interpretation.  I think this should be mentioned more prominently, perhaps in the abstract.

*Answer: We are pleased that the reviewer has noted that the objective in this study was to evaluate the policy as we have mentioned this several places and tried to be explicit on this throughout the manuscript. This is also specified in the objective in the abstract. Additionally, we specify under limitation that we lack fruit and vegetable consumption data (under limitations: “We also lacked information on consumption of the FFV which may have enhanced interpretation and translation of our findings”). Additionally, under implications and further work in the discussion we mention that inclusion of fruit and vegetable consumption in future studies designed to evaluate nationwide policies may aid in understanding potential mechanisms (page 25, lines 605-607).*

7) "Parental education was used as an indicator for socioeconomic position. We used the highest parental education  (from either mother or father) prior to the policy exposure when the children were four years  old."  This seems like a crude measure of SES, especially since the measure was binary (higher+ vs. <higher).  It seems like the measure of geographic location may also be crude, although I don't know enough about Norway to assess this with confidence.  Only four locations were considered: North; Mid; West; and South-East.  I would expect substantial variation in population characteristics by location within these large geographic regions.  Categorization of populations into urban, semi-urban, and rural seems reasonable.

*Answer: Our response is merged with the response to comment #8 below as they are better answered together.*

8) Concerns about possible confounding are heightened by the following: "Allocation of the FFV policy could not be considered 'as if' random. Combined (FFV) schools are more likely to be in areas of lower population density compared to pure elementary (NFFV) schools and are thus more common in rural regions of Norway  such as the North (see S4 Text and S5 Table)"  Table 1 also indicates differences in region and education level between FFV and NFFV groups.  The authors adjust for measured confounders but clearly the populations being compared are not the same.  The authors acknowledge this and attempt to adjust for it, but I think they are perhaps too confident that the conclusions are not affected by it, given the limitations of how possible confounders were measured and categorized.

*Answer: We agree with the reviewer’s point about dichotomization of the parental education level used as an indicator for SES. We have done additional analyses where parental education is split into several categories. This was done to (a) check the robustness of the main results and (b) to gain an insight into the potential for residual confounding by socio-economic position. This is described in the supporting information and results are tabulated (see S6 Text and S10 Table). The results did not provide any indication that bias from inappropriate control for socio-economic confounding would materially alter our conclusions.*

*Socio-economic differences are also captured and adjusted for indirectly in our study, e.g. they reflect onto where a person lives (location and population density) and most importantly on the pre-intervention BMI and trajectory. We agree that some confounding is still likely to exist but given the checks and triangulation we performed we still think this is unlikely to have been sufficient to alter our conclusions.*

*The measure of geographic location (by region) is crude, however, Norway is a small country with about 5.4 million people and this regional division is commonly used in a Norwegian setting. Nevertheless, even when we use county (subdivided into 14 levels) instead of region to adjust for location, the results were similar. We have included a comment on this in Supporting information S12 Table. We also adjusted for population destiny, which adds another layer of control to any geographical differences that may exist between those exposed and unexposed to the FFV policy.*

*Finally, we should note while social inequalities in health in Norway are particularly associated with education, health inequalities in Norway are relatively low across most of the socio-economic distribution compared to other countries (Norwegian Institute of Public Health and Strand et al. ^[3]^). So although there were geographical differences and socio-economic differences (parental education) in the exposure to FFV policy, the confounding effect of these differences is probably much smaller than would be expected in a population with steeper socio-economic gradients such as the US.*

9) In figure 1, I believe the bottom two pooled graphs are mislabeled as boys.

*Answer: The reviewer is correct. Thank you for pointing this out. We have corrected this error.*

10) Figure 6 is nice.  I like seeing the full distributions.  It's somewhat hard to see the lines though.

*Answer: Thank you. The lines are close together because of the small difference in effect, however we have hopefully made the lines easier to see with the color changes.*

11) The discussion says (509-512) "A two-year follow-up evaluation of the FV program in Arkansas US, showed a mean  0.17 z-score reduction in BMI among children exposed to the FFV program compared to  strictly matched unexposed children, and also a three percentage point reduction in  school-level obesity as a result of the program [8]. We found no evidence to support such a  benefit."  This is accurate, but unless I misunderstood something, the current study may not have been able to detect an effect size of this magnitude (especially the older cohort with longer exposure).  If baseline OB/OW prevalence is 40% and F/V reduced it to 37%, that's an OR of 0.88 ((40/60) / (37/63)), which appears to be within or close to the 95% CIs of the current study.  So it may be misleading to frame this as a failure of replication.  I think this is doubly true if one considers that the 95% CI of the two estimates probably overlap quite a bit.  This point should be discussed.  I see that in the Arkansas study, the sample size of the intervention group was not especially large, but they went to greater lengths to match intervention and control subjects.  This may have yielded greater power to detect a smaller effect.

*Answer: The reviewer correctly addresses an important point, which we agree with as stated in our answer above. We have therefore revised our sentence to: “... program [8]. While the confidence intervals from our pooled results overlap with their findings, we observed little evidence to support such a benefit in our sample” (page 21, lines 522-523).*

*Nonetheless, there are key differences in the population, study design and analysis between our study and the Arkansas study (Qian et al., 2016^[4]^) which may explain why we reach different conclusions. First, the US FFV program targeted low-income children which is markedly different from the nationwide Norwegian FFV policy (targeting all children). Rates of obesity are almost 10 times higher in Arkansas vs Norway and the Arkansas US study reported a 3% reduction in obesity rates, not overweight rates as we studied. We believe this is an important distinction between the two evaluations which we also mention in our discussion (page 21-22, lines 525-530). Second, while both studies could not assume “as if” randomization of the intervention, schools self-selected into the US FFV program whereas there was no self-selection in Norwegian schools. The confounding structure caused by this and the socio-economic and ethnic gradients in outcomes between the two populations is thus likely to be very different. Third, the propensity score method used for matching and hence to control for confounding was different to ours. The Qian et al. (2016) paper started with a sample size of 48,637 (treatment group n=1116). After matching with the strictest method, the sample size was 335 and most of the treatment group were not matched (n =162/1116). The characteristics of the analysis sample were also radically different, for example, the proportion of black people in the original sample was 21% in the control pool and 14.8% in the intervention pool, after matching this was 4.1% and 3.8% respectively. In our study we have tried to understand the strength, direction, and main causes of confounding by using a sequence of models and through sensitivity and secondary analyses to triangulate the findings. We have also used a modelling approach that uses all available data under reasonable assumptions about the missing data, to minimize selection bias. Through this multi-faceted approach, we were able to comment on the likely biases, their extent, and their implications.*

*Matching will increase precision for a given sample size, however, it is necessary to have a reasonably large set of variables to generate a predictive model for treatment to match with, which was not available in our study. The matched approach also answers a different question, it targets the effect in a population who were eligible for treatment (i.e., the matched sets). Our approach asks the question what would happen if the whole population were treated (as captured in our nationwide sample) regardless of the propensity for intervention – this is how national FFV policies are implemented and so we think this is the key question. We have added a comment regarding this in the text: “Further, the matched analysis in the study from Arkansas US addresses a different question, it seeks the policy-effect in those eligible for the intervention, ours is concerned with the policy-effect in the whole population.” (page 21-22, lines 527--530).*

Thank you,

Stephan J Guyenet

**Reviewer #2:** Manuscript PMEDICINE-D-21-04001R1 with title "No strong evidence of benefits or unintended consequences on BMI or overweight from a nationwide school fruit and vegetable policy: a quasi-natural experimental study" provides an interesting study on secular trends of overweight among adolescents in Norway and explores whether a free fruit program had any effects. Authors applied very sophisticated and robust modelling for their study. Basically their findings show that having a national policy on school fruit and vegetables does not affect BMI. It is good practice to publish non significant results of public health nutrition interventions.

*Answer: We would like to thank the reviewer for commenting on our paper. We observed little evidence of any practical effect on population BMI or OW/OB, either benefit or unintended consequence. We agree that it is important to publish the evidence from this study, regardless of the direction or lack of, particularly because evidence is lacking and this is a common public health policy.*

What I miss from this paper is a more thorough multidisciplinary discussion. It is a pity that data is already collected, and I am not asking for this, however, it is key to note that it would have benefited from more variables e.g. attitudes or values. Results are not well explained/discussed, although they could have been better explained if e.g. focus groups or interviews would have been performed, or additional consumer-relevant information would have been collected together with the food intake and anthropometric measurements. The confounding in such studies is large, and authors do not mention what kind of recommendations are usual at that age, or the determinants of food choices among adolescents, or the environmental cues in Norway. Have there been any behaviour interventions accompanying the availability change? Was there any special recommendation or social marketing campaigns to support the F&V intake? Authors have not addressed such common sense issues.

Additionally, changing eating behaviour is a huge societal challenge, it is difficult, deserves full attention and it should be addressed in a comprehensive manner. This paper simply shows that increasing availability of fruits and vegetables does not affect body weight, which is somehow expected, but it fails to provide a more in depth consideration of the many other factors that affect food choice.

*Answer: We agree with the reviewer about the benefits of having additional variables, such as attitudes, values, actual consumption of fruit and vegetables, etc. We acknowledge that we lack this. We have changed a sentence in our revised manuscript to emphasize this important point under implications and further work in the discussion (page 25, lines 605-607).*

*There are no specific recommendations to children and adolescents at that age deviating from the general nutrition recommendations to the Norwegian population. The policy did not include additional interventions beyond fruit and vegetable provision, which is we now have specified in our text “The FFV policy was not accompanied by other components beyond FV provision” (page 6, lines 138-139). This was a policy solely providing a daily piece of fresh fruit or vegetable to school children.*

*Although there are several other ‘common sense’ issues affecting dietary intake in society, these would likely have affected all children. Our aim of the paper was to evaluate implications of the nationwide policy on weight outcomes. Other interventions and societal changes relevant for our estimation of the effect of the FFV policy is mentioned in supporting information, S11 Table and S7 Text.*

*The reviewer is correct in that we do not provide an in-depth consideration of other factors that affect food choice, thereby possibly affecting OW/OB outcomes among children and adolescents. Changing dietary behavior is complex. Several factors influence overweight and obesity and expecting free school fruit and vegetable provision to yield large effects on OW/OB is probably unlikely. We therefore discuss our results in relation to similar evaluations (page 21-22, lines 519-546) and mention the link between provision of fruit and vegetables and compensatory behavior changes and why this might result in no overall aggregated policy effect (page 23, lines 555-562). We argue we have discussed our results in a relevant way, which is appropriate to supply future research and policy implementation. Nevertheless, we discuss certain aspects of the reviewers’ points when mentioning other studies aimed to improve childhood obesity, affecting other determinants of overweight and obesity, see page 22, lines 538-543. As the reviewer point out, obesity is multifaceted, which we now specify in the discussion (page 24, lines 597-599). We believe an in-depth discussion on additional (complex) factors contributing to dietary intake and OW/OB prevalence beyond free fruit and vegetable availability is somewhat outside the scope of this paper. Our main aim was to evaluate the efficacy of the Norwegian free fruit and vegetable policy on weight outcomes.*

The paper is nice to read, very good specialist material, high level statistics, but lacks comprehensiveness.

*Answer: Thank you, we hope our revision somewhat accommodates a more comprehensive discussion and the importance that future work strives to include additional variables to provide insight into the complexity of human eating behavior and effects on overweight and obesity.*

**Reviewer #3**: This is a well-conducted study on the impact of a nationwide school fruit and vegetable policy on BMI or overweight of school children in Norway. The study design, datasets, and statistical methods and analyses are mostly adequate. The adjustement for confunding factors is key for the design of a quasi-natural experimental study and the authors addressed this well by adjusting adequately for region, population density, cohort, and parental education. However, there are still a few major issues needing attention especially on presentation of the results.

*Answer: Thank you for acknowledging our study as well-conducted and for valuable feedback that has improved this paper.*

1) Overall the presentation of the results is a bit all over the place with too much technical details which became difficult to focus and follow. Many tables and figures could be put into supplementary information and only need briefly summarised in the main text. For example, table 1 is a huge table with too many details which is not very informative. Can either remove table 1 to supplementary or make it concise with key messages. Table 2 on pre-intervention can be remove to supplementary information as it's not key outcome. The same for Figure 3. It's only on a subgroup analysis but huge with many plots so can be removed to supplementary info.

*Answer: Thank you for this comment. With multiple cohorts of different exposure duration, and one cohort only having cross-sectional data after the intervention it was a complex study to present. However, it is this complexity that facilitates the different comparisons that allowed us to triangulate findings and strengthen our conclusions with respect to the policy. In assembling the manuscript we tried to strike a balance between transparency (showing all of the results- hence the use of the forest style plots) and simplicity (ensuring key messages are not lost). It seems we may not have achieved this.*

*As such, in response we have (a) made changes to the text of the manuscript that hopefully helps the reader navigate and understand why so many details are important. Such as specifying this consistently under Methods – Analyses - Main Analysis page 13, lines 344, 350, and 356-357. We also believe S1 Figure simplifies this. (b) moved Table 1 to supporting information, as the text summarizes the key information (now S7 Table). And (c) moved Table 2 to supporting information (now S8 Table) since we recognize that this is a partial duplication of figure 3.*

*For now we have kept figure 3 in the main text because a key assumption for causal inference in these types of interrupted time-series designs is that the pre-intervention slopes are similar (Lopez Bernal, 2018^[5]^), which is also noted in the text under “estimating the free fruit and vegetable policy effect” in the method section and in S3 Text and S3 figure.*

2) Table 1. There are 4 cohorts but why pooled results only with 3 cohorts? Throughout the paper, especially in Figure 2, there are different combination of 3 of 4 cohorts analysed and pooled, which is a bit difficult to follow. Authors need to make the analyses consistent and neat. If not, please explain why and also what's the impact of using different cohorts on the analyses? Any bias or limitation?

*Answer: Thank you for this comment. We had longitudinal data for three of the four cohorts as mentioned under answer to comment #1. To make this clearer in the method section, we included a superscript in Table 1 (now S7 Table) specifying that cohorts 2010, 2015, and 2017 had longitudinal data and were pooled in analysis of BMI_SDS_ and OW/OB. Longitudinal data from the 2012 cohort was not collected due to lack of time and resources in the NCGS. However, waist circumference was collected in 2012 (and 2010 and 2015 but not 2017) hence is was a useful cohort to use. We have described how the cohorts allow for several comparisons to triangulate evidence, investigate biases, and strengthen the causal inference in the beginning of the method section, see page 7, lines 195-200.*

3) The section of Population distributions and also Figure 6 can be removed to supplementary info as not key at all.

*Answer: Figure 6 illustrates how the policy effect-estimates reflect onto the distribution of BMI and waist circumference in the population. We think it is useful to present since it provides a bounded estimate of the potential magnitude of the policy effect based on the information in our study. Although our study provided little evidence against the null, we think that showing this plausible range is useful because although we cannot rule out no effect, we can provide evidence that our study does not support effects outside this range. We also think it is helpful to see the magnitude of any effect in the context of the distribution in the population and in the original raw units i.e. BMI in kg/m^2^. For these reasons and also because of reviewer #1’s favorable comment about this figure, we have decided to keep it in the main text for now. If the reviewer strongly disagrees, we can move it to the supporting information.*

4) The paper could be improved to be more focused and concise with key messages presented. At the moment, the readers are overwhelmed with huge and complex tables and figures and technical details, many of which could be better placed in supplementary information.

At the moment, there are so many outcomes and analyses presented but could authors consider if they can be classified/identified as primary or secondary outcomes so that the paper is focused on the key messages.

*Answer: Please see our response to point #1. We acknowledge that this study is somewhat complex due to four different cohorts, where three have longitudinal data and one has cross-sectional data. As stated above, this complexity allows us to run several sets of comparisons and draw stronger conclusions. There are differences in exposure duration between the cohorts. We believe inclusion and use of all cohorts and both longitudinal and cross-sectional data is argued for as mentioned under comment #1. We have read through the manuscript and done minor edits to make it more clear, focused, and concise. For example, edits to main analysis description (see response to comment #1) and as described above, we have also moved several tables to supporting information.*

*There were only four outcomes (BMI_SDS_, OR of OW/OB, WC, and WHtR) and all are primary outcomes. A priori we did not consider any of the outcomes to be primary or secondary because each provides different information about the weight-related or adiposity related outcomes. For example, in estimating the effect on BMI, we are interested in the shift in location of the distribution, while for OW/OB we are investigating the odds of overweight and obesity at the end of the distribution, finally for WC/WtHR we have a marker of central adiposity which is argued to be used in addition to BMI as it is linked to metabolic risk among school children (McCarthy et al. 2006 and Javed et al. 2015^[6]^). For these reasons, we don’t make any distinction between primary and secondary outcomes. Each outcome provides an important purpose as an adiposity/obesity indicator and gives different information.*

5) Figure 2 is a key figure. As the different cohorts have different time exposoure to FFV policy, have the pooled results been adjusted for this exposure difference in the analyses?

*Answer: We adjusted for cohort in the pooled analyses, which to some extent adjusts for the different time exposure between cohorts. However, an important reason for stratifying by cohort was to reflect the different exposure period of each. Unfortunately, with only 3 or 4 cohorts, 2 of which had the same duration of exposure, it is not possible to disentangle the duration of exposure from an effect that might be explained by cohort differences– see DAG (S4 Fig).*

6) Missing data and children transferred between types of schools. Need a section specifically on the above issues on the details of missingness and ways to deal with the missingness and discussion of potential biases. It seems missing data were simply removed from the analyses but do we know the pattern of the missingness and what is the potential bias by excluding this missing data?

*Answer: We used longitudinal multilevel models to ensure we used all available data (such that all children with at least one outcome measure were included) and minimize selection bias caused by missing data. These models also make softer assumptions about any missing data compared to other commonly used general linear models. We also set up the data for the longitudinal analysis such that each child had no more than one measure in each target age interval, this was done to minimize bias caused by children who were assessed more frequently in the routine health assessments since children are often monitored more frequently for a reason (i.e, introducing a potential missing not at random mechanism). This is explained in the text (under Methods – analytical dataset and in S3 Text).*

*There were actually only a relatively small proportion of children excluded due to missing data. Taking the main pooled longitudinal models for BMI which included cohorts 2010, 2015, and 2017. The maximum available sample size was 8427, of which 7810 (93%) were analyzed. Most of the missing data were in the 2017 cohort where we were unable to ascertain FFV exposure in 317/1907 (16.6%). For the cross-sectional analysis of waist circumference outcomes which used cohorts 2010, 2012, and 2015, 9718/10,028 (97%) were analyzed. This information has now been added to the opening paragraph of the results (page 15, lines 393-400).*

*In the pre-intervention adjusted models some children (15%) were excluded due to having no measure of BMI before the intervention (this is a reflection of the retrospective cohort design of the studies as described in the main text). To assess selection bias due to these exclusions, we refitted the longitudinal models using the sample with information on pre-intervention BMI – this was described in S12 Table in supporting information that summarizes sensitivity and secondary analysis. The results were very similar to the main analysis using all available data. Both sets of results are shown in the Table A below. This suggests that selection of children that had an earlier BMI measure would not bias our results. Given this and our arguments above, it doesn’t seem conceivable that bias caused by missing data would materially affect our conclusions.*

Table A. Pooled model estimates for the FFV policy effect on BMI_SDS_ in the crude and adjusted longitudinal models using all available data (as per the main text) and the sub-sample with a measure of pre-intervention BMI.

|  |  | Using all available data (as in main text) | | | Using sample with a pre-intervention measure of BMI | | |
| --- | --- | --- | --- | --- | --- | --- | --- |
|  |  | n | Policy effect estimate (95% CI)† | p | n | Policy effect estimate (95% CI)† | p |
| Boys | Crude | 3973 | -0.01 (-0.08, 0.06) | 0.84 | 3365 | -0.01 (-0.08, 0.06) | 0.86 |
|  | Adjusted | 3973 | -0.02 (-0.09, 0.05) | 0.60 | 3365 | -0.02 (-0.09, 0.06) | 0.66 |
| Girls | Crude | 3837 | 0.04 (-0.03, 0.11) | 0.29 | 3254 | 0.03 (-0.04, 0.10) | 0.38 |
|  | Adjusted | 3837 | 0.02 (-0.05, 0.09) | 0.65 | 3254 | 0.01 (-0.06, 0.08) | 0.79 |

†Expressed as the difference in outcome for BMI_SDS_ versus the counterfactual (as estimated using the NFFV schools) with 95% CI. models include terms for cohort (intercept and slope). Adjusted model includes region, population density, highest parental education (all intercept and slope).

BMI_SDS_: body mass index standard deviation scores; CI: confidence interval; FFV: free fruit and vegetables; NFFV: no free fruit and vegetables.

**References in responses**

Qian Y, Nayga RM, Thomsen JMR, Rouse HL. The Effect of the Fresh Fruit and Vegetable Program on Childhood Obesity. Applied Economics Perspectives and Policy. 2016;38(2):260-75. doi: <https://doi.org/10.1093/aepp/ppv017>.

Lawlor DA, Tilling K, Davey Smith G. Triangulation in aetiological epidemiology. Int J Epidemiol. 2016 Dec 1;45(6):1866-1886. doi: 10.1093/ije/dyw314.

The rich are outliving the poor in both Norway and USA [Internet]. Norwegian Institute of Public Health. Available from: <https://www.fhi.no/en/news/2019/rich-outliving-the-poor/>

Strand et al. Social inequalities in health [Internet]. Norwegian Institute of Public Health. Available from: <https://www.fhi.no/en/op/hin/groups/social-inequalities/>

Lopez Bernal J, Cummins S, Gasparrini A. The use of controls in interrupted time series studies of public health interventions. Int J Epidemiol. 2018;47(6):2082-93. Epub 2018/07/10. doi: 10.1093/ije/dyy135. PubMed PMID: 29982445.

McCarthy HD, Ashwell M. A study of central fatness using waist-to-height ratios in UK children and adolescents over two decades supports the simple message--'keep your waist circumference to less than half your height'. Int J Obes (Lond). 2006 Jun;30(6):988-92. doi: 10.1038/sj.ijo.0803226. PMID: 16432546

Javed A, Jumean M, Murad MH, Okorodudu D, Kumar S, Somers VK, et al. Diagnostic performance of body mass index to identify obesity as defined by body adiposity in children and adolescents: a systematic review and meta-analysis. Pediatr Obes. 2015;10(3):234-44. Epub 2014/06/26. doi: 10.1111/ijpo.242. PubMed PMID: 24961794.

1. Qian Y, Nayga RM, Thomsen JMR, Rouse HL. The Effect of the Fresh Fruit and Vegetable Program on Childhood Obesity. Applied Economics Perspectives and Policy. 2016;38(2):260-75. doi: <https://doi.org/10.1093/aepp/ppv017>.
2. Lawlor DA, Tilling K, Davey Smith G. Triangulation in aetiological epidemiology. Int J Epidemiol. 2016 Dec 1;45(6):1866-1886. doi: 10.1093/ije/dyw314.
3. # The rich are outliving the poor in both Norway and USA [Internet]. Norwegian Institute of Public Health. Available from: <https://www.fhi.no/en/news/2019/rich-outliving-the-poor/> and Strand et al. Social inequalities in health [Internet]. Norwegian Institute of Public Health. Available from: https://www.fhi.no/en/op/hin/groups/social-inequalities/
4. Qian Y, Nayga RM, Thomsen JMR, Rouse HL. The Effect of the Fresh Fruit and Vegetable Program on Childhood Obesity. Applied Economics Perspectives and Policy. 2016;38(2):260-75. doi: <https://doi.org/10.1093/aepp/ppv017>.
5. Lopez Bernal J, Cummins S, Gasparrini A. The use of controls in interrupted time series studies of public health interventions. Int J Epidemiol. 2018;47(6):2082-93. Epub 2018/07/10. doi: 10.1093/ije/dyy135. PubMed PMID: 29982445.
6. McCarthy HD, Ashwell M. A study of central fatness using waist-to-height ratios in UK children and adolescents over two decades supports the simple message--'keep your waist circumference to less than half your height'. Int J Obes (Lond). 2006 Jun;30(6):988-92. doi: 10.1038/sj.ijo.0803226. PMID: 16432546; and Javed A, Jumean M, Murad MH, Okorodudu D, Kumar S, Somers VK, et al. Diagnostic performance of body mass index to identify obesity as defined by body adiposity in children and adolescents: a systematic review and meta-analysis. Pediatr Obes. 2015;10(3):234-44. Epub 2014/06/26. doi: 10.1111/ijpo.242. PubMed PMID: 24961794.
