# Supplementary material for: A nationwide school fruit and vegetable policy and childhood and adolescent overweight: A quasi-natural experimental study
Source: PLoS Med. 2022 Jan 18;19(1):e1003881. doi: 10.1371/journal.pmed.1003881 (PMC8765663; doi:10.1371/journal.pmed.1003881)
Supplement: Attachment — Submitted filename: Response to editors.docx [file pmed.1003881.s025.docx]

**Response to editors (and reviewers)**

**Requests from Editors:**

Please check and update the URLs in your Data Availability Statement, as both lead to pages that no longer exist.

*Answer: We have updated the URLs.*

In your abstract, please quantify your main findings with 95% CI and p values (where appropriate).

*Answer: Thank you for suggesting this. We have now added the main estimates of the policy effect on BMI, overweight or obesity and waist circumference in boys and girls aged 8.5 years – see lines 47-51. To ensure the abstract is not too unwieldy with results we have removed the statement that reports the population shift in median BMI and WC (see manuscript with tracked changes, line 85) as this seems unnecessary now that we have provided the other estimates.*

Line 52: Please update to ‘The main limitations of this study are the potential for residual confounding and exposure misclassification, despite efforts to minimise their impact on conclusions’.

*Answer: We have made the requested change.*

Line 67: This bullet can be deleted.

*Answer: We have deleted this bullet point.*

Line 69: Please update to ‘To promote a healthy diet, from 2007 to 2014 a nationwide free fruit and vegetable policy ensured a daily piece of free fruit or vegetable was available to all children in Norwegian combined schools (covering grades 1-10)’.

*Answer: We have made the requested change, however included the word “that” in the sentence. The sentence now reads: ‘To promote a healthy diet, from 2007 to 2014 a nationwide free fruit and vegetable policy ensured that a daily piece of free fruit or vegetable was available to all children in Norwegian combined schools (covering grades 1-10, age 6 to 16 years)’.*

Please also consider including the age group covered by Norwegian combined schools, as grade age boundaries will differ between countries. 

*Answer: We agree and have inserted this in the sentence mentioning grades in the abstract and author summary, lines 35 and 72.*

Line 71: Please update to ‘Studies on the potential benefits or consequences of such fruit and vegetable policies are important in improving public health efforts to tackle childhood overweight and obesity’.

*Answer: We have made the requested change.*

Line 81: Delete the sentence beginning ‘Data are from’ and instead relocate this to the start of the following bullet such that it reads ‘Using data from 11215 Norwegian children and early adolescents, we observed little…’

*Answer: We have made the requested change.*

Line 143: ‘We did not register’.

*Answer: We have made the requested change.*

Line 196: Please update to ‘Other data’

*Answer: We have made the requested change.*

In Figures 3 and 5, please use the legend to denote which statistical test was used to derive the p values.

Please also check supplementary figures in light of the comment above.

*Answer: We have changed the figure legends in Fig 3 and 5, and S8, S9, and S11 Figures: “The p-values are a Wald test of the interaction between parental education and FFV/subscription program.”*

Line 578: Update ‘little’ to ‘few’.

*Answer: We have made the requested change.*

Line 661: The ‘Ethics approval’ section can be removed as it is now redundant. 

*Answer: We have deleted this section.*

References 3 and 5: Please include (date accessed: DD/MM/YYYY).

*Answer: We have made inserted date accessed.*

Reference 41 contains unnecessary COI information, please remove this.

*Answer: Thank you for pointing out this error. We have corrected this.*

Please ensure all journal abbreviations in the references are consistent with those found in the National Center for Biotechnology Information (NCBI) databases.

*Answer: We have checked journal abbreviations and hope they now are in accordance with the NCBI databases.*

Please update your STROBE checklist to use section and paragraph numbers as opposed to page numbers (which are liable to change during the publication process).

*Answer: We have updated the STROBE checklist with section and paragraph numbers.*

**Comments from Reviewers:**

Reviewer #1: The authors adequately addressed my concerns.  I agree with the authors that post-hoc power analysis would not be informative.  Thank you,

Stephan J Guyenet

*Answer: Thank you for providing helpful comments improving our manuscript.*

Reviewer #3: Thanks authors for their great effort to improve the manuscript. I am satisfied with the response and revision. No further issues needing attention.

*Answer: Thank you for providing helpful comments improving our manuscript.*
